# A deep learning framework for accurate reaction prediction and its application on high-throughput experimentation data

**DOI:** 10.1186/s13321-023-00732-w

**Published:** 2023-08-11

**Authors:** Baiqing Li, Shimin Su, Chan Zhu, Jie Lin, Xinyue Hu, Lebin Su, Zhunzhun Yu, Kuangbiao Liao, Hongming Chen

**Affiliations:** Guangzhou Laboratory, Guangzhou, 510005 Guangdong China

## Abstract

In recent years, it has been seen that artificial intelligence (AI) starts to bring revolutionary changes to chemical synthesis. However, the lack of suitable ways of representing chemical reactions and the scarceness of reaction data has limited the wider application of AI to reaction prediction. Here, we introduce a novel reaction representation, GraphRXN, for reaction prediction. It utilizes a universal graph-based neural network framework to encode chemical reactions by directly taking two-dimension reaction structures as inputs. The GraphRXN model was evaluated by three publically available chemical reaction datasets and gave on-par or superior results compared with other baseline models. To further evaluate the effectiveness of GraphRXN, wet-lab experiments were carried out for the purpose of generating reaction data. GraphRXN model was then built on high-throughput experimentation data and a decent accuracy (R^2^ of 0.712) was obtained on our in-house data. This highlights that the GraphRXN model can be deployed in an integrated workflow which combines robotics and AI technologies for forward reaction prediction.

**Supplementary Information:**

The online version contains supplementary material available at 10.1186/s13321-023-00732-w.

## Introduction

Organic synthesis is the foundation for the development of life science, such as pharmaceutics and chemical biology [1, 2]. For decades, the discovery of chemical reaction was driven by serendipitous intuition stemming from expertise, experience and mechanism exploration [3]. However, professional chemists sometimes have hard time to predict whether a specific substrate can indeed go through a desired reaction transformation, even for some well-established reactions [4, 5]. When optimizing reaction yield or selectivity, small changes in reaction factors, including catalysts, temperature, ligands, solvents, and additives, may result in outcomes that deviate from the intended target.

With the development of artificial intelligence (AI), computational methods to predict the reaction outcome and retro-synthesis route have been proposed to accelerate chemical research [6–12]. There is a rich history of computer assisted chemical synthesis. Jorgensen and coworkers introduced Computer Assisted Mechanistic Evaluation of Organic Reactions (CAMEO [13]). This and other early approaches, including SOPHIA [14] and Robia [15], attempted to employ expert heuristics to define possible mechanistic reactions. What these approaches suffered in common is the difficulties to enable prediction of novel chemistry. For specific reaction classes with sufficiently detailed reaction condition data, machine learning can be applied to the quantitative prediction of yield [16]. As a sub-domain of AI, deep learning technologies were booming in the last decade and have made huge impact on reaction prediction and retro-synthesis modelling. For retro-synthesis planning, there are two types of deep learning model. One type is the so called template-based models, where combining reaction templates with deep neural networks [17–19] has been applied. Reaction templates are the classic approach to codifying the “rules” of chemistry [20–23], and is extensively applied in computer-aided synthesis planning [24, 25]. In contrast, without using any pre-defined reaction templates, various deep learning based machine translation models were employed to learn chemical reaction from data directly and can also been used for synthesis planning. That is called template-free-based model.

For the prediction of reaction outcome, Quantum-mechanics (QM) based descriptors, representing electrostatic or steric characterizations, calculated by density functional theory (DFT) or other semi-empirical methods [26–29] are frequently used for modelling. Doyle et al*.* [16] utilized QM-derived descriptors to build a random forest model, which achieved good prediction performance of the Buchwald-Hartwig cross-coupling of aryl halides with 4-methylaniline. Sigman et al. [30] defined four important DFT parameters to capture the conformational dynamics of the ligands, which were fed into multivariate regression modelling for the correlation of ligand properties and relative free energy. Denmark et al. [10] generated a set of three-dimension QM descriptors to develop an AI-based model for enantioselectivity prediction. Applying QM descriptors to modelling offers the advantage of model interpretability, but it usually requires a deep understanding of reaction mechanisms, which may be difficult to transfer to other reaction prediction tasks. Another kind of popular descriptors is the so-called reaction fingerprints. Glorius and co-workers [31] developed a multiple fingerprint features (MFFs) as molecular descriptors, by concatenating 24 different fingerprints, to predict the enantioselectivities and yields for different experimental datasets. Although good results were observed, this method can be a time and resource intensive process, as a single molecule was represented in a 71,374-bit array. Reymond et al. [32] reported a molecular fingerprint called differential reaction fingerprint (DRFP), by taking reaction SMILES as input which were embedded into an arbitrary binary space via set operations for subsequent hashing and folding, to perform reaction classification and yield prediction. Though the reaction fingerprints are easily built, the reaction fingerprint may lose certain chemical information due to the limited predefined substructures, and thus a task-specific representation which could learn from dataset is needed.

One possible solution to the issue of universal reaction descriptors is to apply graph neural networks (GNNs) on reaction prediction task [33, 34]. Owing to the powerful capacity for modelling graph data, GNNs have recently become one of the most popular AI methods and have achieved remarkable prediction performance on several tasks [11, 35–37]. Various graph-based models, such as graph conventional network(GCN) [11, 38], GraphSAGE [39], graph attention network(GAT) [40] and message passing neural network(MPNN) [41], have been proposed to learn a function of the entire input graph over molecular properties, by either directly applying a weight matrix on the graph structure or using a message passing and aggregation procedure to update node features iteratively. A molecule is regarded as a graph, where atoms are treated as nodes and bonds are treated as edges. Node and edge features are influenced by proximal ones, and these features are learned and aggregated to form the embedding of entire molecule graph [41, 42]. It was worth mentioning that in addition to the above mentioned graph model architectures, transformer neural network [43] was adopted for the direct processing of molecular graph as sets of atoms and bonds [44, 45]. For example, transformer based model *GraphormerMapper* [46] was proposed to do reaction featurization, which is similar to the idea of learning molecular graph features with reaction data, but based on a transformer architecture.

In this work, we proposed a modified communicative message passing neural network (GraphRXN), which was used to generate reaction embeddings for reaction modelling without using predefined fingerprints. For chemical reactions comprised of multiple components, reaction features can be built up by aggregating embeddings of these components together and correlated to the reaction output via a dense layer neural network.

Another major challenge for reaction prediction is the access of high-quality data [47, 48]. Though numerous data were accumulated, bias toward positive results in the literatures led to unbalanced datasets. What’s more, extracting valid large-scale data from literature requires substantial human intervention. High-throughput experimentation (HTE) is a technique that can perform a large number of experiments in parallel [49, 50]. HTE could serve as a powerful tool for advancing AI chemistry as it has the capability to significantly increase experiment throughput, and ensure data integrity and consistency. With this technology, several high-quality reaction datasets were reported [47], including Buchwald-Hartwig amination [16, 51, 52], Suzuki coupling [9, 53, 54], photoredox-catalysed cross coupling [55]. These datasets contain both successful and failed reactions, which is critical for building forward reaction prediction models. Three public HTE datasets were used as proof of concept studies for our method and encourage results were demonstrated. As further verification, we used our in-house HTE platform to generate data of Buchwald-Hartwig cross-coupling reaction. The GraphRXN methodology was then applied on the in-house dataset and a decent prediction model was obtained (R^2^ of 0.713), which highlights that our method can be integrated with reaction robotics system for reaction prediction. We expect that deep learning based methods like GraphRXN, combined with the data-on-demand reaction machine, could potentially push the boundary of reaction methodology development [56, 57].

## Methods

### GraphRXN framework

**Fig. 1:**
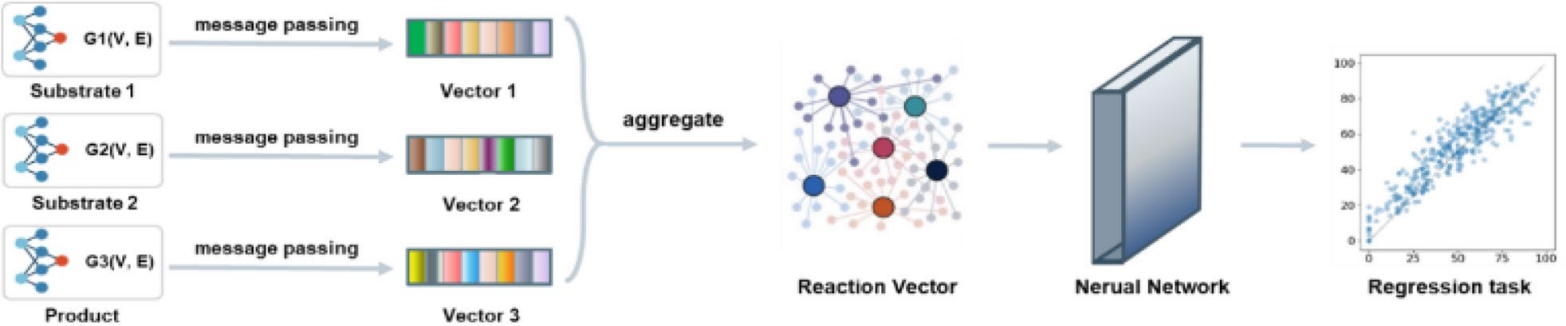
Model architecture of GraphRXN

A deep-learning graph framework, GraphRXN, was proposed to be capable of learning reaction features and predicting reactivity (Fig. 1).

The input of GraphRXN is the reaction SMILES where each reaction component (either reactants or products) is represented by the directed molecular graph ${\varvec{G}}({\varvec{V}},{\varvec{E}})$ [58]. For each individual graph of the reaction, it learns through three steps, including message passing, information updating, and read out. All node features (${{\varvec{X}}}_{v}, \forall v\in {\varvec{V}}$*)* and edge features** (**${{\varvec{X}}}_{{e}_{v,w}},\forall {e}_{v,w}\in {\varvec{E}}$) are propagated in the message passing and updating stage as shown in Algorithm 1:

- for the $v$ node at step *k*, its intermediate message vector ${m}^{k}\left(v\right)$ is obtained by aggregating the hidden state of its neighbouring edges at the previous step ${h}^{k-1}\left({e}_{u,v}\right)$, then the previous hidden state ${h}^{k-1}\left(v\right)$ is concatenated with its current message ${m}^{k}\left(v\right)$ and fed into a communicative function to obtain current node hidden state ${\mathrm{h}}^{k}\left(v\right)$;
- for the edge ${e}_{v,w}$ at step *k*, its intermediate message vector ${m}^{k}\left({e}_{v,w}\right)$ is obtained by subtracting the previous edge hidden states ${h}^{k-1}\left({e}_{v,w}\right)$ from hidden state of its starting node ${h}^{k}\left(v\right)$, then the initial edge state ${h}^{0}\left({e}_{v,w}\right)$ and weighted vector $\mathrm{W}\bullet {m}^{k}\left({e}_{v,m}\right)$ are added up and fed into an activation function ($ReLU$) to form current edge state ${h}^{k}\left({e}_{v,w}\right)$;
- After $K$ steps iteration, the message vector ($m\left(v\right))$ is obtained by aggregating hidden states ${h}^{K}\left({e}_{u,v}\right)$ of its neighbouring edges. The node message vector $m\left(v\right)$, current node hidden state ${h}^{K}\left(v\right)$ and initial node information $x\left(v\right)$ are fed into a communicative function to form the final node embedding $h\left(v\right)$.
- Gated Recurrent Unit (GRU) is chosen as the readout operator to aggregate the node vectors into a graph vector. The length of molecule feature vector is adjustable (here it is set to 300 bit). 
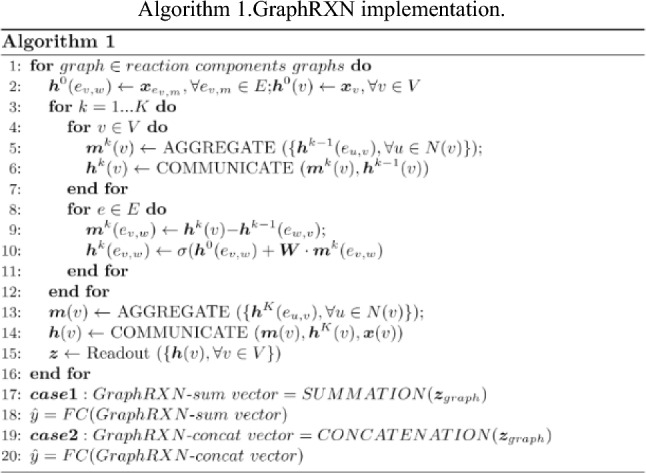

The molecular feature vectors are then aggregated into one reaction vector by either summation or concatenation operation (named as GraphRXN-sum and GraphRXN-concat respectively). The length of GraphRXN-sum vector is set to 300 bit and GraphRXN-concat is multiple times of 300 (depending on the maximal reaction components). If we take a two-components reaction (A + B → P), for example, when summation operation is selected to aggregate features of A, B and P, the length of reaction vector is 300 bit; when concatenation operation is selected to aggregate molecular features, the length of reaction vector is 900 bit. In addition, for some reaction components which are inappropriate to be depicted by graph structure, such as inorganic reagents or catalysts, one-hot embedding will be used to characterize them. Finally, a dense layer is used to fit reaction outcomes, including reaction yield and selectivity.

### Data preparation

As shown in Table 1, in total, four reaction datasets were used to validate the performance of our GraphRXN model. Three of them are open-source HTE datasets and one of them is generated from in-house HTE platform (in Additional file 1).

**Table 1:** Description of reaction datasets. Dataset 1–3 were available public datasets, and dataset 4 was generated by our in-house HTE platform

Entry	Description	Size	Source
Dataset 1	Yield for Buchwald-Hartwig coupling reaction	4608	Doyle et al. [16]
Dataset 2	Yield for Suzuki–Miyaura coupling reaction	5760	Perera et al. [53]
Dataset 3	Stereo-selectivity for asymmetric N, S-acetal formation reaction	1075	Denmark et al. [10]
Dataset 4	Ratio for Buchwald-Hartwig coupling reaction	1558	In-house HTE dataset

The original outcome value $x$ (including yields, selectivities, and ratios) was then treated with z-score normalization, where $\mu$ is the mean of all samples, $\upsigma$ is the standard deviation of all samples.

1 $$\hat{x} = \frac{x - \mu }{{\upsigma }}$$

Each dataset was split into training set and test set in a ratio of 80:20. To be mentioned, a validation set (20% of training set) was raised to avoid overfitting, *i.e.* when the model performance on validation set became stable, the training process would stop. From the *k*-fold cross validation (CV) task, we obtained averaged errors, rather than depending on one randomly split. To make a strict comparison, ten folds CV was adopted on dataset 1–2 which was consistent with the reported Yield-BERT study by Reymond et al. [8, 59], and dataset 3 which was consistent with the reported study by Perera et al. [53]. Five folds CV was adopted in the in-house dataset.

### Baseline models

Two previously published reaction prediction methods Yield-BERT [8, 59] and DeepReac +  [12] were used as baseline models for comparison.

1. Yield-BERT is a sequence-based model which employ natural processing architecture to predict reaction related properties given a text-based representation of the reaction, using an encoder transformer model combined with a regression layer. The source codes of Yield-BERT were downloaded from https://rxn4chemistry.github.io/rxn_yields/.
2. DeepReac + is also a graph based model. In terms of model architecture, unlike the message passing neural network used in GraphRXN, DeepReac + employed GAT model, a variant of GNN, as the core building block. The source codes of DeepReac + were downloaded from https://github.com/bm2-lab/DeepReac.

Hyper-parameters search and minor modifications were conducted for resolving some incompatibility issues of python environment. Other training details about four models (including hyper-parameters selection and training log) were supplemented in part 2 in supplementary materials.

### Model evaluation

GraphRXN method along with two baseline models were applied on all four datasets. Regarding the performance measures, three evaluation metrics on the test set were used, including correlation coefficient (R^2^), mean absolute error (MAE) and root mean squared error (RMSE).

### HTE platform

**Fig. 2:**
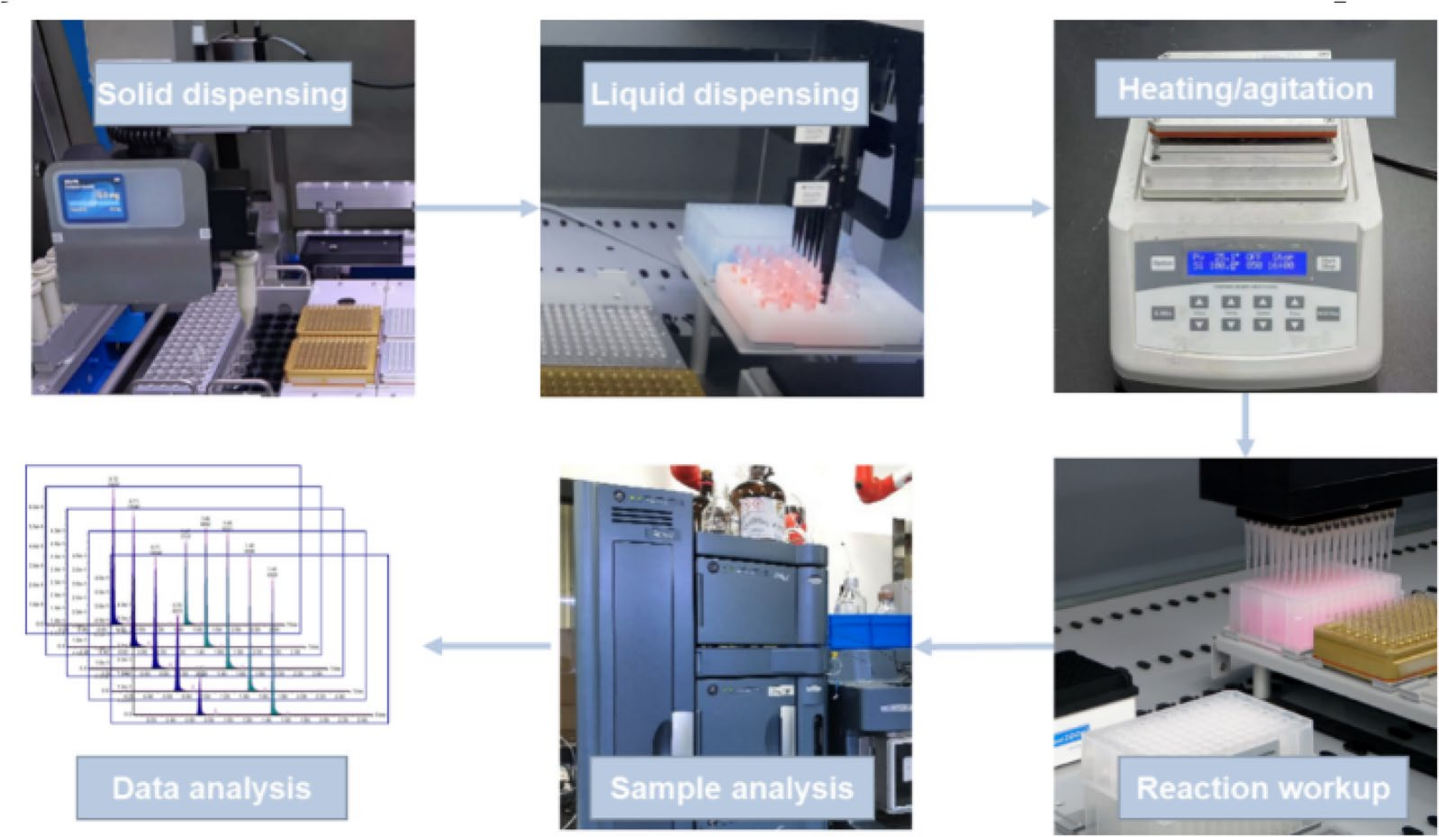
General workflow of HTE process

HTE, operated under standard codes, has been used to perform parallel experiments for rapid screening arrays of reactants or conditions, which generated large amounts of high-quality reaction data [60, 61]. We have developed an in-house HTE platform by assembling various state-of-the-art automated workstations/modules. All experiments in this study were carried out using HTE, including solid dispensing, liquid dispensing, heating and agitation, reaction workup, sample analysis and data analysis (Fig. 2). Exquisite design of experiment was required before THE [62].

### Solid dispensing

Solid samples were stored in the dispensing containers. Then an overhead gravimetric dispensing unit delivered target amounts of samples from dispensing containers to the designated 4 mL vials.

### Liquid dispensing

Liquid samples were stored in uniform bottles. Then the liquid-handling robot transferred target volume of samples to the designated 4 mL vials in a programmed manner. With the amounts of solid and liquid samples dispensed in 4 mL vials, the liquid-handling robot was used again to make stock solution accordingly. All stock solutions were mixed thoroughly using vortex mixer. Stock solutions were transferred into the designated glass tubes of 96-well aluminium blocks for reaction setup using the liquid-handling robot.

### Heating and agitation

The 96-well aluminium blocks were placed on orbital agitators under pre-set temperature and time.

Reaction workup: After the reactions were stopped and cooled down, pipetting workstation was used to process the reaction mixtures in batches, including quenching, dilution and filtration. Then samples were prepared in 96-well plates for UPLC-MS analysis.

### Sample analysis

Samples were sequentially injected into UPLC-MS for expected substance determination and quantification.

### Data analysis

Raw data generated by UPLC-MS were fed into Peaksel [63], an analytical software developed by Elsci, which was capable of executing batch-level integration rendering us the UV response area of target substance.

### Experimental preparation

**Fig. 3:**
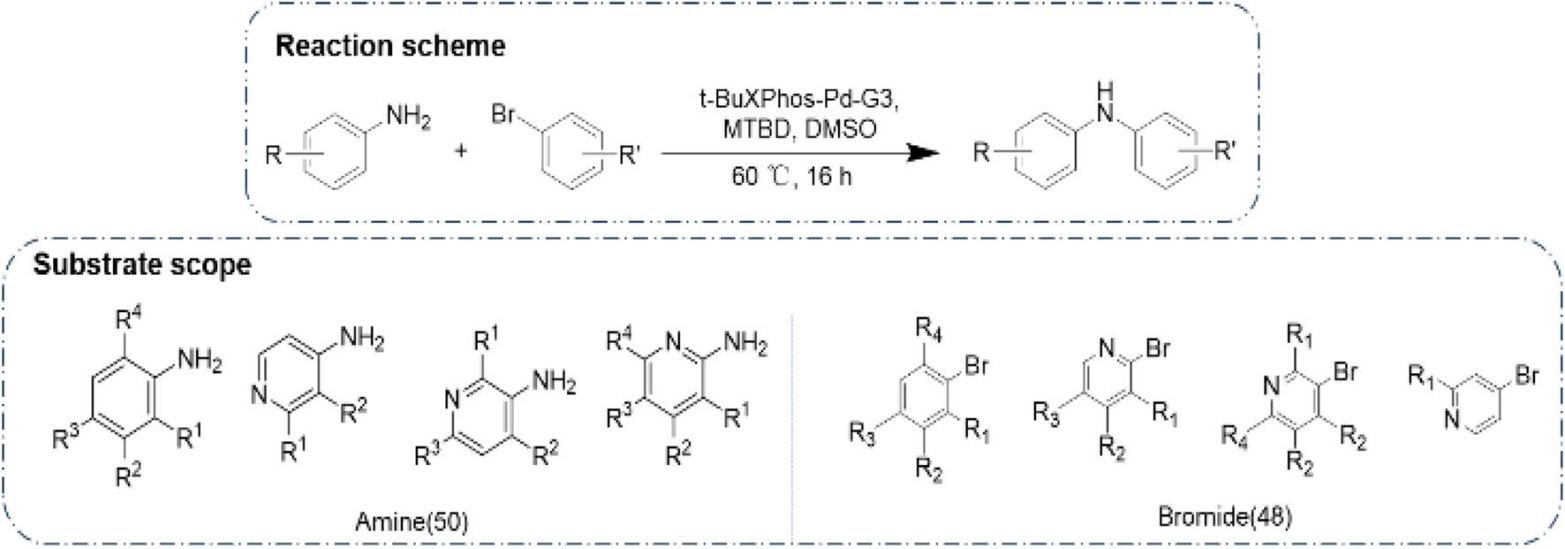
Reaction scheme and substrate scope

Buchwald-Hartwig coupling reaction was used as examined reaction in this study, to further evaluate GraphRXN on the in-house dataset as further verification (Fig. 3).

For the standard condition, we used t-BuXPhos-Pd-G3 as catalyst, 7-Methyl-1,5,7-triazabicyclo[4.4.0]dec-5-ene (MTBD) as base, and DMSO as solvent (Fig. 3a). Firstly, the palladium precatalyst t-BuXPhos-Pd-G3 collocate with MTBD performed well with primary amines [64–66]. Secondly, the catalyst and base are DMSO soluble which would facilitate the HTE process [67, 68].

As of substrates, a series of *ortho-*, *meta-*, and *para*-substituted, including electron-donating and electron-withdrawing groups, aryl-Br and aryl-NH_2_ were selected (Fig. 3b). In total, 50 primary amines (26 Ph-NH_2_, 24 Py-NH_2_) and 48 bromides (24 Ph-Br, 24 Py-Br) were used in our dataset generation (see Additional file 2: Figs. S1–S2).

### Experimental workflow on HTE platform

In this study, all reactions were carried out at 0.016 mmol scale in 96-well aluminum blocks using HTE platform. For reaction setup, all robots were embedded in a glovebox filled with N_2_. The 96-well aluminum blocks were sealed under N_2_ and then subjected to orbital agitators with the pre-set parameter of 850 rpm and 65 °C. After 16 h, the 96-well aluminum blocks were cooled down to room temperature. In total, 2127 reactions were successfully conducted on HTE platform (detailed HTE layout sees part1.4 in supplementary materials).

For each glass tube, 0.0625 equivalence of 4,4ʹ-Di-tert-butyl-1,1ʹ-biphenyl was added as internal standard (IS). Reaction solutions were then transferred to filter plates and the filtrates were collected by 96-well plates. The sample plates were then analyzed by UPLC-MS. The UV responses of product and IS were obtained using Peaksel. The ratios of UV response of product over IS (${ratio}_{UV}$) were calculated using the following equation, where ${A}_{product}$ is the response area of the target product at the wave length of 254 nm,${A}_{IS}$ is the response area of the IS at the wave length of 254 nm, $c$ is a constant (0.0625 eq.), which represents the mole ratio of IS and product at 100% theoretical yield:

2 $$ratio_{UV} = \frac{{A_{product} \times c}}{{A_{IS} }} \times 100{\text{\% }}$$

During the course of data analysis, 569 reaction data derived from abnormal spectra were discarded. Eventually, 1558 reaction data were obtained.

For more details about the experiments, please see part 1 in supplementary materials.

## Results

### Performance on public datasets

Four models, including Graph-concat, Graph-sum, Yield-BERT and DeepReac + , were built on three public datasets. Dataset 1 and 2 are collections of reaction yield from coupling reactions, while Dataset 3 is a collection of stereo-selectivity from asymmetric reactions. The average R^2^, MAE and RMSE values for the respective test set throughout the tenfold CV procedure are listed in Table 2.

**Table 2:** Comparison of model performance on public dataset 1–3. The values of R^2^, MAE, RMSE refers to the mean and standard deviation across the folds

Dataset	Methods	R^2^	MAE	RMSE
Dataset 1	GraphRXN-concat	0.951 ± 0.004	4.3 ± 0.1	6.0 ± 0.2
	GraphRXN-sum	0.938 ± 0.006	4.9 ± 0.2	6.8 ± 0.3
	Yield-BERT	0.951 ± 0.005	4.0 ± 0.2	6.0 ± 0.3
	DeepReac +	0.922 ± 0.019	5.2 ± 0.6	7.5 ± 0.9
Dataset 2	GraphRXN-concat	0.844 ± 0.007	7.9 ± 0.1	11.1 ± 0.3
	GraphRXN-sum	0.838 ± 0.009	8.1 ± 0.2	11.3 ± 0.4
	Yield-BERT	0.815 ± 0.013	8.1 ± 0.4	12.1 ± 0.5
	DeepReac +	0.827 ± 0.017	8.1 ± 0.4	11.7 ± 0.6
Dataset 3	GraphRXN-concat	0.892 ± 0.008	0.16 ± 0.01	0.23 ± 0.01
	GraphRXN-sum	0.881 ± 0.013	0.18 ± 0.01	0.24 ± 0.02
	Yield-BERT	0.886 ± 0.010	0.16 ± 0.01	0.24 ± 0.01
	DeepReac +	0.853 ± 0.024	0.18 ± 0.01	0.25 ± 0.02

For Dataset 1, the performance of GraphRXN-concat model (R^2^ of 0.951) was similar to the baseline method Yield-BERT (R^2^ of 0.951), but better than the GraphRXN-sum (R^2^ of 0.937) and DeepReact + (R^2^ of 0.922) models. For Dataset 2, both GraphRXN-concat (R^2^ of 0.844) and GraphRXN-sum (R^2^ of 0.838) outperformed the Yield-BERT (R^2^ of 0.815) and DeepReact + (R^2^ of 0.827) method. For Dataset 3, the R^2^ of GraphRXN-concat was 0.892, which was better than GraphRXN-sum (0.881), Yield-BERT (0.886) and DeepReac + (0.853). Among these three metrics, we believe that MAE is more meaningful for chemists, as it gives a possible error between the observed and predicted values. MAE/RMSE may better serve as a reference value for chemists to decide whether to conduct the experiment or not. Our GraphRXN-concat model gave better MAE and RMSE values than Yield-BERT and DeepReac + , which demonstrated that GraphRXN model can provide on-par or slightly better performance over the baseline models. Details of model prediction on each fold were included in Additional file 2: Tables S6–S8.

### HTE results

**Fig. 4:**
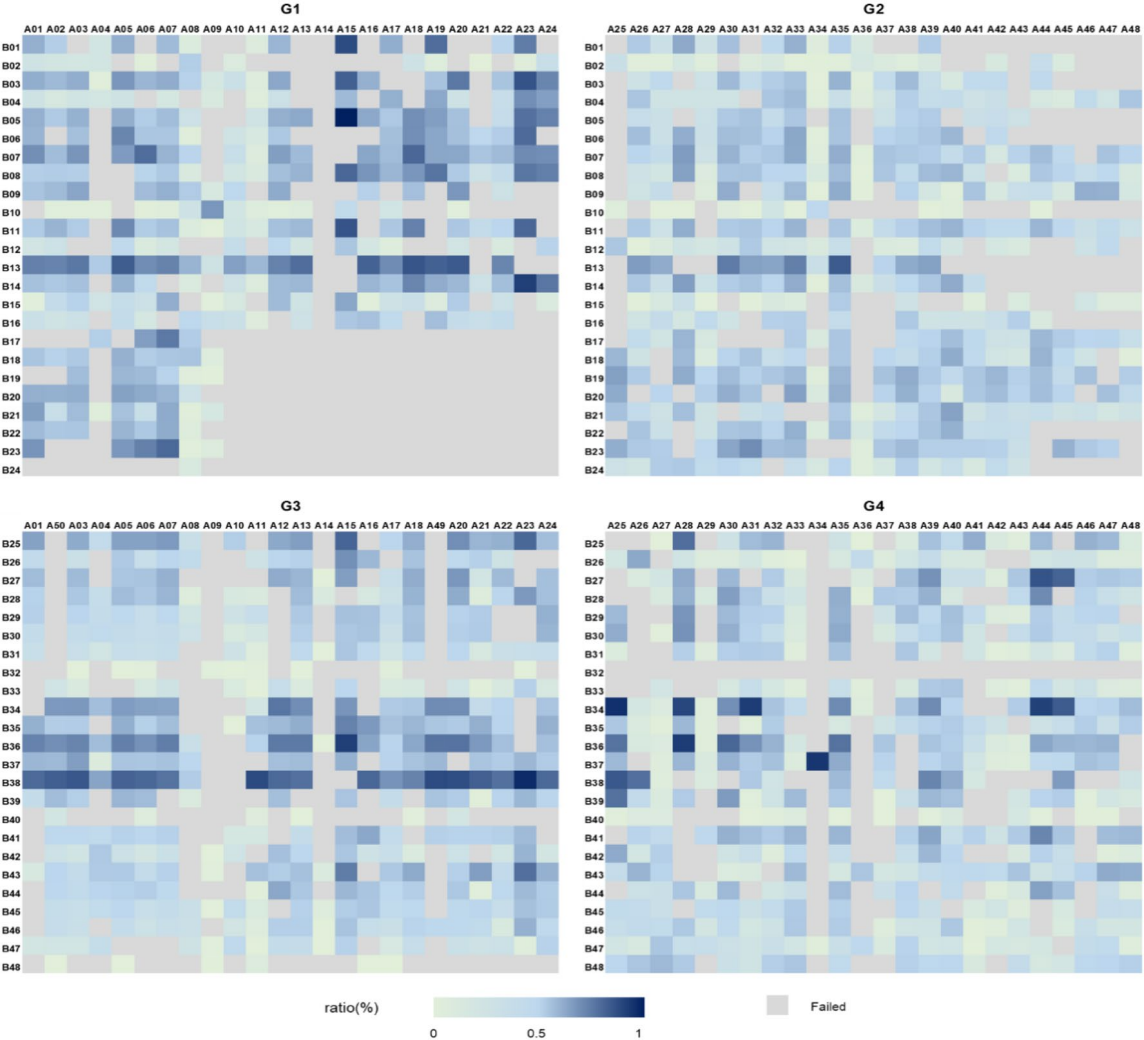
Heatmap of ${Ratio}_{UV}$ distribution for the in-house reaction dataset, where the prefix “**A**” in X-axis represents amine, and the prefix “**B**” in Y-axis represents bromide

Wet-lab experiment was conducted in this study, and 1558 data points were collected into the ultimate dataset (See Additional file 1). According to the substituted aromatic amines/bromides of reactants, reactions can be grouped into four groups (G1-G4), *i.e.* diphenylamines derivatives (reactions between Ph-NH_2_ and Ph-Br, G1), phenylpyridine amine derivatives (reactions between Ph-NH_2_ and Py-Br, G2), phenylpyridine amine derivatives (reactions between Py-NH_2_ and Ph-Br, G3) and 2,2ʹ-dipyridylamide derivatives (reactions between Py-NH_2_ and Py-Br, G4). G1 contains 317 reaction points, while G2, G3 and G4 group have 419, 401 and 421 reactions respectively. Hereby shows the ${ratio}_{UV}$ distribution for all four groups, where the light color represents low value, and the dark color corresponds to high value, ranging from 0 to 1 (Fig. 4). The grey grids represent failed reactions or discarded data and the data filtering policy were supplemented in part 1.6 of supplementary materials.

For the entire dataset, half of the reaction ratio lies in the range from 0 to 0.2. The ${ratio}_{UV}$ distribution was not balanced with heavy condense on low value which would be a challenging task for modelling. Among these, 13% of reactions in G1 gave ratio ≥ 0.5, while only 0.7%, 8% and 5% for G2, G3 and G4 respectively, which indicates the chosen reaction condition in HTE may be more suitable for reactions between Ph-NH_2_ and Ph-Br.

### Performance on in-house HTE dataset

**Fig. 5:**
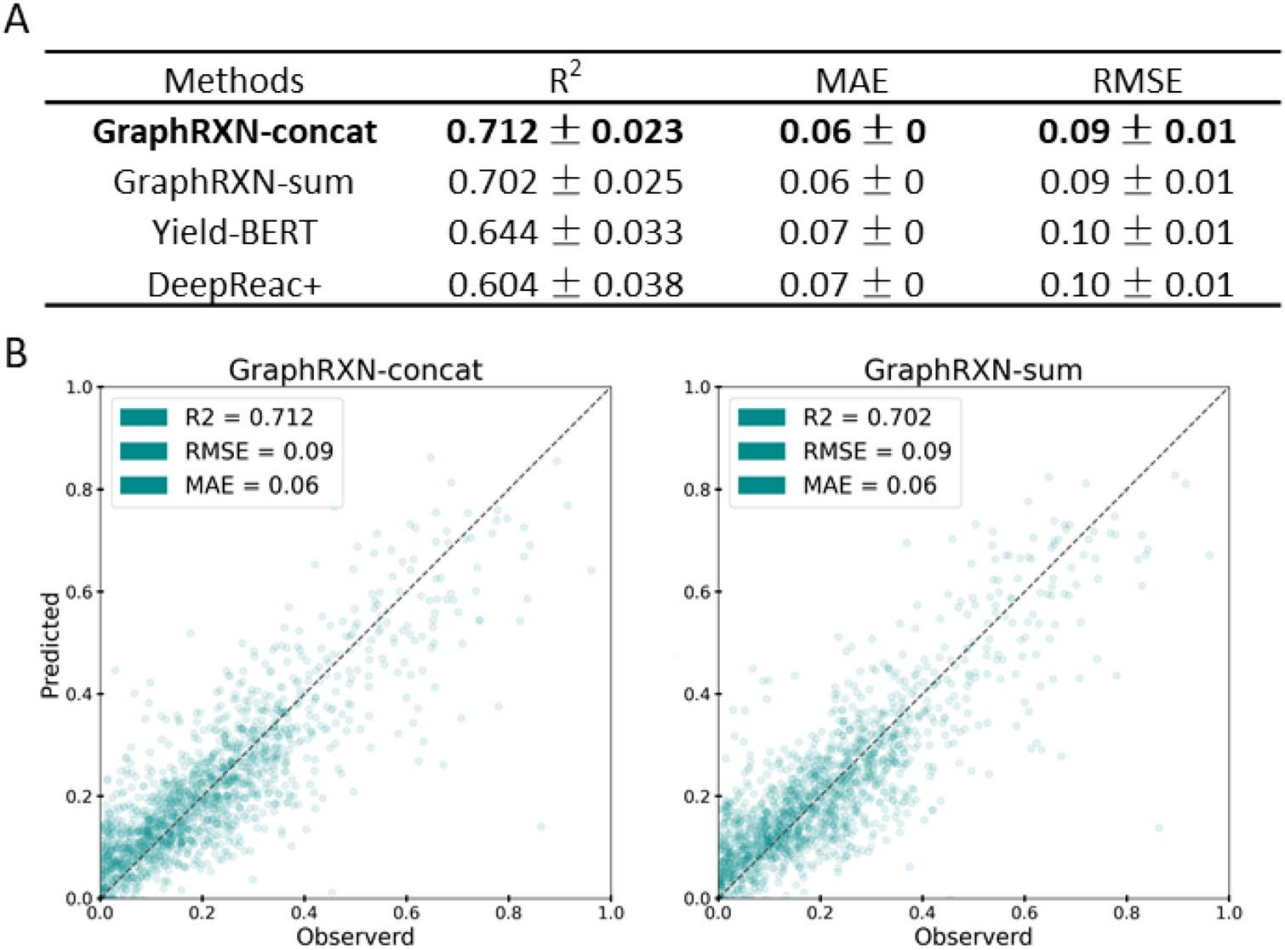
Results of GraphRXN and other baseline models on in-house HTE dataset. **A** evaluation metrics over five-fold CV on test set. **B** test set plots over five-folds CV of GraphRXN-concat and GraphRXN-sum

An in-house dataset of 1558 data points was used for modelling and five-fold CV without replacement was done for train-test split. Results of GraphRXN and other baseline model are shown in Fig. 5. Our GraphRXN model obtained better performance on the entire dataset comparing with other baseline methods (R^2^ of 0.712, MAE of 0.06, and RMSE of 0.09 in GraphRXN-concat). Besides, GraphRXN-concat performed slightly better than GraphRXN-sum model on this regression task. Results of each CV fold on test set see Additional file 2: Table S9.

### Performance on scarce data

It is well known that deep learning relies on large amounts of data to discover the relationship between variables and outcomes, data scarcity remains a challenging problem in modelling processes in certain areas, especially in the field of reaction prediction. Here, we discussed the stability on these four aforementioned deep learning methods when handling scarce data.

Four groups of the in-house dataset (G1-G4), which hold smaller size than other published datasets, were evaluated respectively. The performances of GraphRXN and other baseline models are listed in Table 3 and results of each CV fold on test set see Additional file 2: Tables S10–S13. The performances of GraphRXN-concat were superior than other models on G2, G3 and but slightly worse on G1 and G4. It seems that R^2^ on small-size dataset can fluctuate considerably, *e.g.* R^2^ of four groups are rather different from each other, while values of MAE and RMSE are similar across all four groups. The results indicate that the smaller dataset with limited structural diversity that might deteriorate the prediction accuracy, while a larger dataset with diverse structures can allow to learn a better model from a larger reaction space. In general, GraphRXN-concat showed superior or on-par performance on handling scarce data, compared to other deep learning methods.

**Table 3:** Comparison of model performance over four separate reaction groups of our in-house dataset. The values of R, MAE, RMSE refers to the mean and standard deviation across the folds

Group	Size	Methods	R^2^	MAE	RMSE
G1	317	GraphRXN-concat	0.653 ± 0.085	0.08 ± 0.01	0.11 ± 0.01
		GraphRXN-sum	0.453 ± 0.145	0.11 ± 0.01	0.14 ± 0.02
		**Yield-BERT**	**0.712 ± 0.070**	**0.07 ± 0**	**0.10 ± 0.01**
		DeepReac +	0.544 ± 0.128	0.09 ± 0.01	0.13 ± 0.02
G2	419	**GraphRXN-concat**	**0.628 ± 0.048**	**0.05 ± 0**	**0.07 ± 0.01**
		GraphRXN-sum	0.590 ± 0.034	0.06 ± 0	0.07 ± 0
		Yield-BERT	0.512 ± 0.046	0.06 ± 0	0.08 ± 0.01
		DeepReac +	0.523 ± 0.059	0.06 ± 0	0.08 ± 0
G3	401	**GraphRXN-concat**	**0.800 ± 0.030**	**0.06 ± 0**	**0.08 ± 0**
		GraphRXN-sum	0.773 ± 0.020	0.06 ± 0	0.08 ± 0
		Yield-BERT	0.783 ± 0.012	0.06 ± 0	0.08 ± 0
		DeepReac +	0.744 ± 0.032	0.07 ± 0.01	0.09 ± 0.01
G4	421	GraphRXN-concat	0.445 ± 0.088	0.08 ± 0.01	0.12 ± 0.01
		GraphRXN-sum	0.405 ± 0.091	0.09 ± 0.01	0.12 ± 0.01
		**Yield-BERT**	**0.490 ± 0.055**	**0.08 ± 0.01**	**0.11 ± 0.01**
		DeepReac +	0.208 ± 0.17	0.10 ± 0.01	0.14 ± 0.02

Bold emphasis represents the best model performance in each group

### Variable-length graph representation

**Fig. 6:**
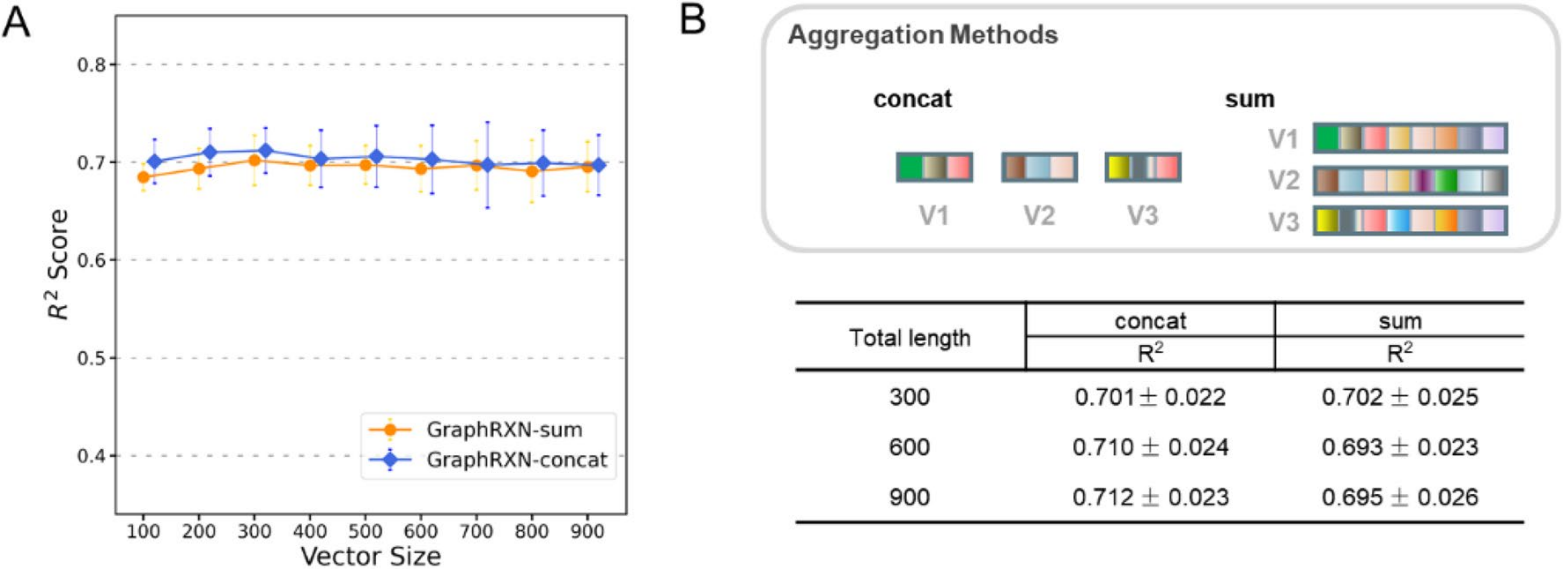
**A** Variance of model performance with different vector size. Vector size ranges from 100 to 900 bit, where 100 bit as the interval. **B** Model performance when using either concatenation or summation to construct reaction vectors

Our GraphRXN algorithm can provide the variable-length representation that are relevant to each task at hand. Usually, a good representation should be small but dense enough to contain a wealth of information for downstream modelling [69]. Thus, we compared the model accuracy over different size of learned feature, regardless of other aspects of modelling (Fig. 6A). As the vector size climbs from 100 to 900 bits, the results of GraphRXN-concat and GraphRXN-sum remain steady at around 0.7 points. This diagram points out that vector size only caused subtle changes in model performance. Additionally, GraphRXN-concat still provided the higher accuracy in different vector size. The curves reached a peak at the size of 300, which may indicate that the number of 300 should be a suitable size for representation in the molecule level. Detailed values of evaluation metrics were supplemented in Additional file 2: Table S14.

### Aggregation methods for reaction vector

Model processing was sensitive to the ordering of vectors [69], and different order of vectors would render different results, although all else being equal. In this study, two aggregation methods were utilized to encode the reaction vector when graph representation was ready. Specific order must be set in concatenating reaction vectors, and for example, in this study, we used the vector order as *aromatic amines, bromides and products*. In this way, we assumed it would be a possible way to sum all components’ vectors together, to eliminate the effect of the input order. We then compared two aggregation methods in the same total length (Fig. 6B). When downstream model took over the same length of reaction vectors, GraphRXN-concat still provided the higher accuracy, except when 100-bit vector is unable for molecules to contain complete information. The explanation of this issue is that summing all the vectors up may weaken the ability of representation bit-wisely, and neglect the relationship between reaction components. According to the existing results, concatenation would be more suitable for characterizing chemical reactions.

## Conclusion

In this work, GraphRXN, a novel computational framework, is proposed to assist automation of chemical synthesis. Regardless of the reaction mechanism, GraphRXN directly takes the 2D molecular structures of organic components as input and learn the task-related representations of chemical reaction automatically during training and achieves on-par or slightly better performance over the baseline models. In addition, we used HTE platform to build standardized dataset, and GraphRXN also delivered good correlations. Although a chemical reaction goes through certain transitional states, it seems that the model can directly predict reaction outcome using structural features of reaction components without the guidance of mechanism. This study has demonstrated that deep learning model could yield moderate to good accuracy in reaction prediction regardless of limited size of the datasets and many complex influencing variables. These results have motivated us to apply this HTE + AI strategy to enable cost reduction and liberate the scientific workforce from repetitive tasks in the future. The source code of GraphRXN and our in-house reaction dataset are available online.

### Supplementary Information

**Additional file 1.** The table of Dataset 4. It consists of 1558 in-house data points about Buchwald-Hartwig coupling reaction.

**Additional file 2.** The supporting materials. It contains the details about the in-house HTE experimental process, model hyperparameters and model porformance.

## Data Availability

All source code and datasets used to produce the reported results can be found at https://github.com/jidushanbojue/GraphRXN.

## References

[1] Campos KR, Coleman PJ, Alvarez JC (2019). The importance of synthetic chemistry in the pharmaceutical industry. Science.

[2] Whitesides GM (2015). Reinventing chemistry. Angew Chemie Int Ed.

[3] Davies IW (2019). The digitization of organic synthesis. Nature.

[4] Raccuglia P, Elbert KC, Adler PDF (2016). Machine-learning-assisted materials discovery using failed experiments. Nature.

[5] Lin S, Dikler S, Blincoe WD (2018). Mapping the dark space of chemical reactions with extended nanomole synthesis and MALDI-TOF MS. Science.

[6] Vaucher AC, Zipoli F, Geluykens J (2020). Automated extraction of chemical synthesis actions from experimental procedures. Nat Commun.

[7] Skoraczyński G, Dittwald P, Miasojedow B (2017). Predicting the outcomes of organic reactions via machine learning: are current descriptors sufficient?. Sci Rep.

[8] Schwaller P, Vaucher AC, Laino T, Reymond JL (2021). Prediction of chemical reaction yields using deep learning. Mach Learn Sci Technol.

[9] Beker W, Roszak R, Wołos A (2022). Machine learning may sometimes simply capture literature popularity trends: a case study of heterocyclic Suzuki-miyaura coupling. J Am Chem Soc.

[10] Zahrt AF, Henle JJ, Rose BT (2019). Prediction of higher-selectivity catalysts by computer-driven workflow and machine learning. Science.

[11] Coley CW, Jin W, Rogers L (2019). A graph-convolutional neural network model for the prediction of chemical reactivity. Chem Sci.

[12] Gong Y, Xue D, Chuai G (2021). DeepReac+: deep active learning for quantitative modeling of organic chemical reactions. Chem Sci.

[13] Salatin TD, Jorgensen WL (1980). Computer-assisted mechanistic evaluation of organic reactions. 1 Overview. J Org Chem.

[14] Satoh H, Funatsu K (1995). SOPHIA, a knowledge base-guided reaction prediction system—utilization of a knowledge base derived from a reaction database. J Chem Inf Comput Sci.

[15] Socorro IM, Taylor K, Goodman JM (2005). ROBIA: a reaction prediction program. Org Lett.

[16] Ahneman DT, Estrada JG, Lin S (2018). Predicting reaction performance in C-N cross-coupling using machine learning. Science.

[17] Coley CW, Barzilay R, Jaakkola TS (2017). Prediction of organic reaction outcomes using machine learning. ACS Cent Sci.

[18] Wei JN, Duvenaud D, Aspuru-Guzik A (2016). Neural networks for the prediction of organic chemistry reactions. ACS Cent Sci.

[19] Segler MHS, Waller MP (2017). Neural-symbolic machine learning for retrosynthesis and reaction prediction. Chem A Eur J.

[20] Corey EJ, Jorgensen WL (1976). Computer-assisted synthetic analysis. Synthetic strategies based on appendages and the use of reconnective transforms. J Am Chem Soc.

[21] Law J, Zsoldos Z, Simon A (2009). Route designer: a retrosynthetic analysis tool utilizing automated retrosynthetic rule generation. J Chem Inf Model.

[22] Christ CD, Zentgraf M, Kriegl JM (2012). Mining electronic laboratory notebooks: analysis, retrosynthesis, and reaction based enumeration. J Chem Inf Model.

[23] Szymkuć S, Gajewska EP, Klucznik T (2016). Computer-assisted synthetic planning: the end of the beginning. Angew Chemie Int Ed.

[24] Coley CW, Green WH, Jensen KF (2018). Machine learning in computer-aided synthesis planning. Acc Chem Res.

[25] Segler MHS, Preuss M, Waller MP (2018). Planning chemical syntheses with deep neural networks and symbolic AI. Nature.

[26] Werth J, Sigman MS (2020). Connecting and analyzing enantioselective bifunctional hydrogen bond donor catalysis using data science tools. J Am Chem Soc.

[27] Werth J, Sigman MS (2021). Linear regression model development for analysis of asymmetric copper-Bisoxazoline catalysis. ACS Catal.

[28] Zahrt AF, Rose BT, Darrow WT (2021). Computational methods for training set selection and error assessment applied to catalyst design: guidelines for deciding which reactions to run first and which to run next. React Chem Eng.

[29] Henle JJ, Zahrt AF, Rose BT (2020). Development of a computer-guided workflow for catalyst optimization. descriptor validation, subset selection, and training set analysis. J Am Chem Soc.

[30] Zhao S, Gensch T, Murray B (2018). Enantiodivergent Pd-catalyzed C-C bond formation enabled through ligand parameterization. Science.

[31] Sandfort F, Strieth-Kalthoff F, Kühnemund M (2020). A structure-based platform for predicting chemical reactivity. Chem.

[32] Probst D, Schwaller P, Reymond J-L (2022). Reaction classification and yield prediction using the differential reaction fingerprint DRFP. Digit Discov.

[33] Zhou J, Cui G, Hu S (2020). Graph neural networks: a review of methods and applications. AI Open.

[34] Wu Z, Pan S, Chen F (2021). A comprehensive survey on graph neural networks. IEEE Trans Neural Networks Learn Syst.

[35] Schwaller P, Vaucher AC, Laplaza R (2022). Machine intelligence for chemical reaction space. WIREs Comput Mol Sci.

[36] Louis S-Y, Zhao Y, Nasiri A (2020). Graph convolutional neural networks with global attention for improved materials property prediction. Phys Chem Chem Phys.

[37] Feinberg EN, Sur D, Wu Z (2018). PotentialNet for molecular property prediction. ACS Cent Sci.

[38] Torng W, Altman RB (2019). Graph convolutional neural networks for predicting drug-target interactions. J Chem Inf Model.

[39] Hamilton WL, Ying R, Leskovec J (2017) Inductive representation learning on large graphs. Adv Neural Inf Process Syst. 1025–1035

[40] Sacha M, Błaż M, Byrski P (2021). Molecule edit graph attention network: modeling chemical reactions as sequences of graph edits. J Chem Inf Model.

[41] Gilmer J, Schoenholz SS, Riley PF (2017). Neural message passing for quantum chemistry. 34th Int Conf Mach Learn ICML.

[42] Schütt KT, Arbabzadah F, Chmiela S (2017). Quantum-chemical insights from deep tensor neural networks. Nat Commun.

[43] Vaswani A, Shazeer N, Parmar N, et al (2017) Attention is all you need. Advances in Neural Information Processing Systems 2017-Decem:5999–6009

[44] Min E, Chen R, Bian Y (2022). Transformer for graphs: an overview from architecture perspective. arXiv.

[45] Dwivedi VP, Bresson X (2020). A generalization of transformer networks to graphs. arxiv.

[46] Nugmanov R, Dyubankova N, Gedich A, Wegner JK (2022). Bidirectional graphormer for reactivity understanding: neural network trained to reaction atom-to-atom mapping task. J Chem Inf Model.

[47] Kearnes SM, Maser MR, Wleklinski M (2021). The open reaction database. J Am Chem Soc.

[48] Baldi P (2021). Call for a public open database of all chemical reactions. J Chem Inf Model.

[49] Shevlin M (2017). Practical high-throughput experimentation for chemists. ACS Med Chem Lett.

[50] Krska SW, DiRocco DA, Dreher SD, Shevlin M (2017). The evolution of chemical high-throughput experimentation to address challenging problems in pharmaceutical synthesis. Acc Chem Res.

[51] Kashani SK, Jessiman JE, Newman SG (2020). Exploring homogeneous conditions for mild Buchwald-Hartwig amination in batch and flow. Org Process Res Dev.

[52] Boström J, Brown DG, Young RJ, Keserü GM (2018). Expanding the medicinal chemistry synthetic toolbox. Nat Rev Drug Discov.

[53] Perera D, Tucker JW, Brahmbhatt S (2018). A platform for automated nanomole-scale reaction screening and micromole-scale synthesis in flow. Science.

[54] Reizman BJ, Wang YM, Buchwald SL, Jensen KF (2016). Suzuki-Miyaura cross-coupling optimization enabled by automated feedback. React Chem Eng.

[55] Kariofillis SK, Jiang S, Żurański AM (2022). Using data science to guide aryl bromide substrate scope analysis in a Ni/Photoredox-Catalyzed cross-coupling with acetals as alcohol-derived radical sources. J Am Chem Soc.

[56] Keith JA, Vassilev-Galindo V, Cheng B (2021). Combining machine learning and computational chemistry for predictive insights into chemical systems. Chem Rev.

[57] Shen Y, Borowski JE, Hardy MA (2021). Automation and computer-assisted planning for chemical synthesis. Nat Rev Methods Prim.

[58] Song Y, Zheng S, Niu Z, et al (2020) Communicative Representation Learning on Attributed Molecular Graphs. In: Proceedings of the Twenty-Ninth International Joint Conference on Artificial Intelligence. International Joint Conferences on Artificial Intelligence Organization, California. 2831–2838

[59] Schwaller P, Probst D, Vaucher AC, Nair VH, Kreutter D, Laino T, Reymond JL (2021). Mapping the space of chemical reactions using attention-based neural networks. Nat Mach Intell.

[60] Isbrandt ES, Sullivan RJ, Newman SG (2019). High throughput strategies for the discovery and optimization of catalytic reactions. Angew Chemie Int Ed.

[61] Tu NP, Dombrowski AW, Goshu GM (2019). High-throughput reaction screening with nanomoles of solid reagents coated on glass beads. Angew Chemie Int Ed.

[62] Cook A, Clément R, Newman SG (2021). Reaction screening in multiwell plates: high-throughput optimization of a Buchwald-Hartwig amination. Nat Protoc.

[63] *Peaksel.* Elsci. https://elsci.io/peaksel/index.html. Accessed 24 July 2023

[64] Surry DS, Buchwald SL (2011). Dialkylbiaryl phosphines in Pd-catalyzed amination: a user’s guide. Chem Sci.

[65] Baumgartner LM, Dennis JM, White NA (2019). Use of a droplet platform to optimize Pd-Catalyzed C-N coupling reactions promoted by organic bases. Org Process Res Dev.

[66] Bruneau A, Roche M, Alami M, Messaoudi S (2015). 2-aminobiphenyl palladacycles: the “most powerful” precatalysts in C-C and C-heteroatom cross-couplings. ACS Catal.

[67] Brocklehurst CE, Gallou F, Hartwieg JCD (2018). Microtiter plate (MTP) reaction screening and optimization of surfactant chemistry: examples of Suzuki-Miyaura and Buchwald-Hartwig cross-couplings in water. Org Process Res Dev.

[68] Gesmundo NJ, Sauvagnat B, Curran PJ (2018). Nanoscale synthesis and affinity ranking. Nature.

[69] Staker J, Marques G, Dakka J, Cartwright HM (2020). Chapter 15. Representation Learning in Chemistry. Machine learning in chemistry: the impact of artificial intelligence.

