# Supplementary material for: A deep learning framework for accurate reaction prediction and its application on high-throughput experimentation data
Source: J Cheminform. 2023 Aug 11;15:72. doi: 10.1186/s13321-023-00732-w (PMC10422736; doi:10.1186/s13321-023-00732-w)
Supplement: Supplementary file 2 — Additional file 2. The supporting materials. It contains the details about the in-house HTE experimental process, model hyperparameters and model porformance. [file 13321_2023_732_MOESM2_ESM.docx]

*Supplementary Materials*

**A deep learning framework for accurate reaction prediction and its application on high-throughput experimentation data**

Baiqing Li ^a, †^_,_ Shimin Su ^a, †^, Chan Zhu ^a^, Jie Lin^a^, Xinyue Hu^a^, Lebin Su^a^, Zhunzhun Yu^a^, Kuangbiao Liao^a^*^, *^*, Hongming Chen^a^*^, *^*

^a^ Guangzhou Laboratory, Guangzhou 510005, Guangdong Province, China

^†^ These authors contributed equally

* Correspondence e-mail: chen_hongming@gzlab.ac.cn; liao_kuangbiao@gzlab.ac.cn

**Contents**

[**Contents** 2](#_Toc135689609)

[**1.General experimental details** 3](#_Toc135689610)

[1.1 Materials and instruments 3](#_Toc135689611)

[1.2 Substrate scope 3](#_Toc135689612)

[1.3 Standard workflow 5](#_Toc135689613)

[1.4 Plate layout 5](#_Toc135689614)

[1.5 Reaction workup 7](#_Toc135689615)

[1.6 Data analysis 7](#_Toc135689616)

[2.Details for modelling process 9](#_Toc135689617)

[2.1 GraphRXN 9](#_Toc135689618)

[2.2 Yield-BERT 14](#_Toc135689619)

[2.3 DeepReac+ 15](#_Toc135689620)

[2.4 Comparison of training details across four models 15](#_Toc135689621)

[3.Details for model performance 16](#_Toc135689622)

[3.1 Prediction performance on public datasets 16](#_Toc135689623)

[3.2 Prediction performance on in-house dataset 18](#_Toc135689624)

[3.3 Model performance under different vector sizes 20](#_Toc135689625)

[4. Reference 21](#_Toc135689626)

**1.General experimental details**

## 1.1 Materials and instruments

Reagents are commercially obtained by Bide, Energy, Macklin, TCI, Acros, Alfa Aesar, and Sigma-Aldrich were used as received. Catalysts, and bases were stored in glovebox (O_2_ less than 5ppm). All reagents are dissolved in stock solution for reaction screening. Stock solution of bromide (0.4mmol/mL in DMSO), amine (0.4mmol/mL in DMSO), base (0.8mmol/mL in DMSO), catalyst (0.04mmol/mL in DMSO) were prepared.

Reactions were analyzed using a Waters Acquity UPLC. Column: Acquity UPLC BEH C18 1.7 μm 2.1 × 50 mm (Part No. 186002350), Mobile Phase A: 0.05% formic acid + Water, Mobile Phase B: MeCN, Weak Wash: 10% MeCN + 90% Water. The instrument was equipped with an SQ Detector 2 with electrospray ionization (ESI) source in the positive mode. Liquid was handled with Eppendorf continuous manual dispenser and/or pipetting with Tecan pipetting device.

## 1.2 Substrate scope

In this part, 50 primary amines (26 Ph-NH_2_, 24 Py-NH_2_) and 48 bromides (24 Ph-Br, 24 Py-Br) used for in-house reaction development were shown below. Chart S1 show the structure of aryl-NH_2_ (A01-A50), and Chart S2 show the structure of aryl-Br (B01-B48).

### Chart S1. Structure of aryl-NH_2_ (A01- A50).


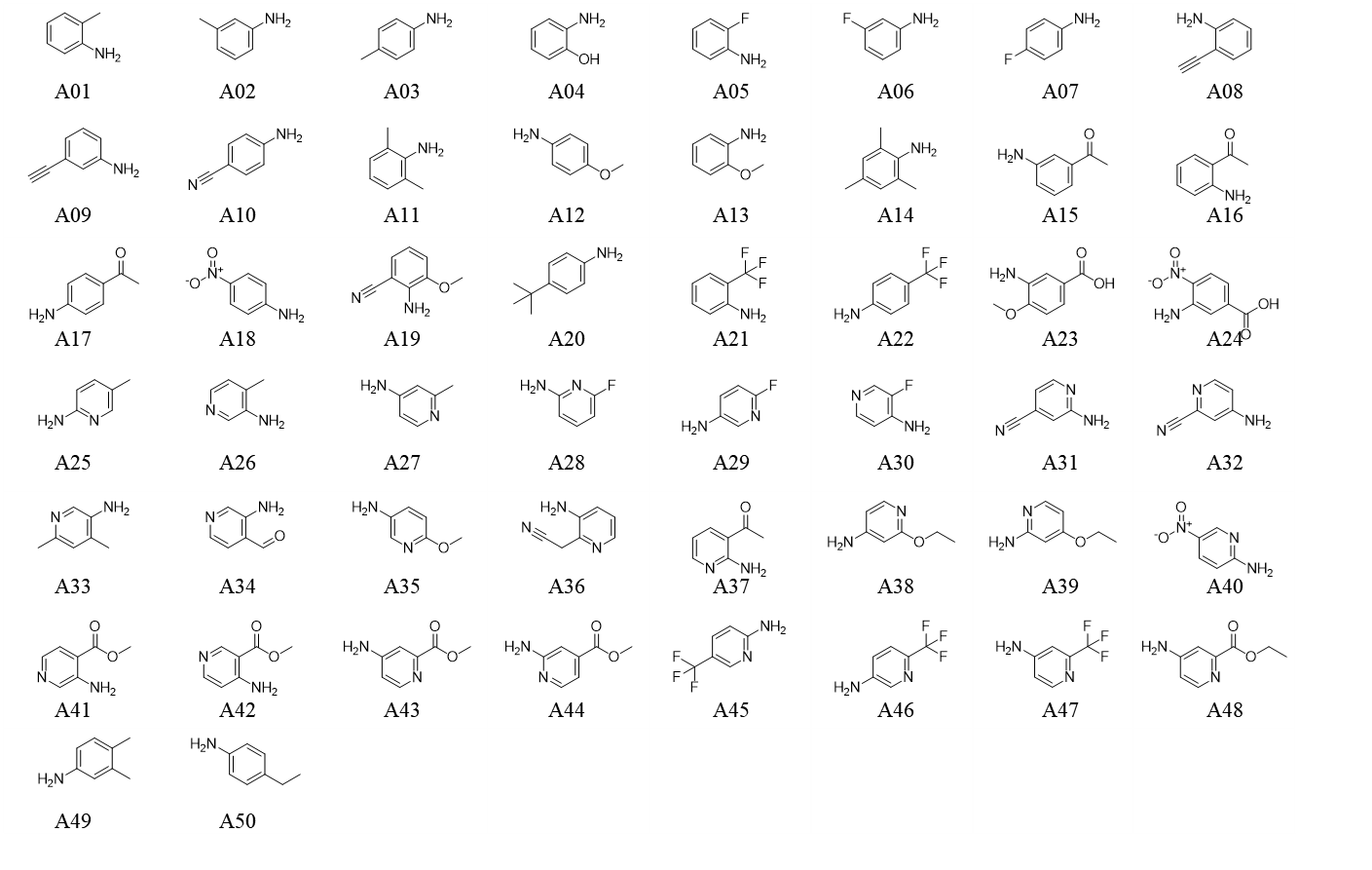


### Chart S2. Structure of aryl-Br (B01-B48).


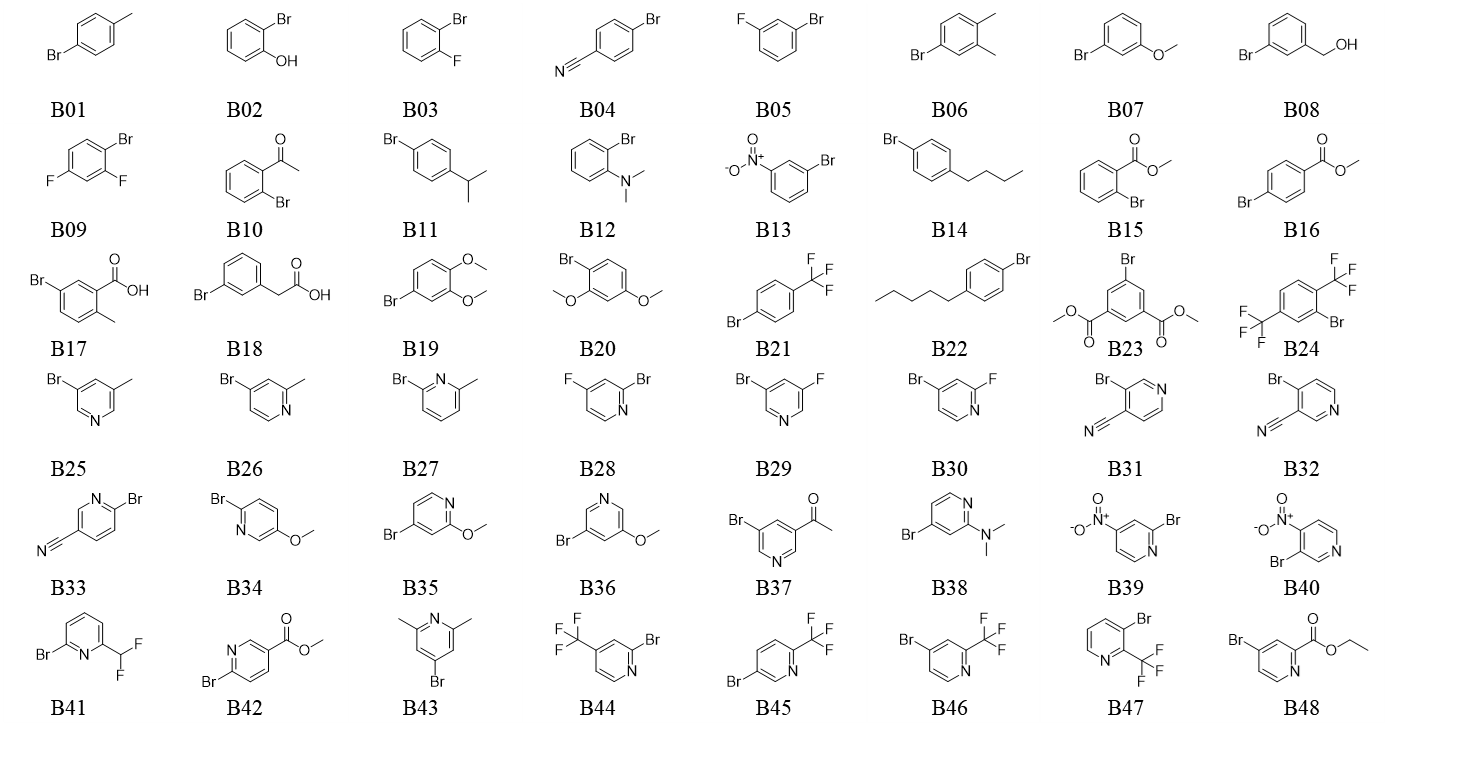


## 1.3 Standard workflow

All reactions were set up inside Vigor glovebox with constant N_2_ purge (oxygen typically < 5 ppm). 96 empty 900μL glass tubes were equipped in 96-well plate. Then, bromide (b01-b48), amine (a01-a50), base, and catalyst were added following the programed codes using liquid handling robot. A control reaction (reaction between a03 and b21) will perform in one of the glass tubes per plate. After finishing liquid transferring, the plate was sealed under N_2_ and the reaction mixture was stirred at 130 ºC for 16 hours.

## 1.4 Plate layout

For each plate, stock solutions were added according to the encodings of amines in column order, and the encodings of bromides in row order. Figure S1 describe general reaction setup and Table S1 describe detailed layout for each plate.


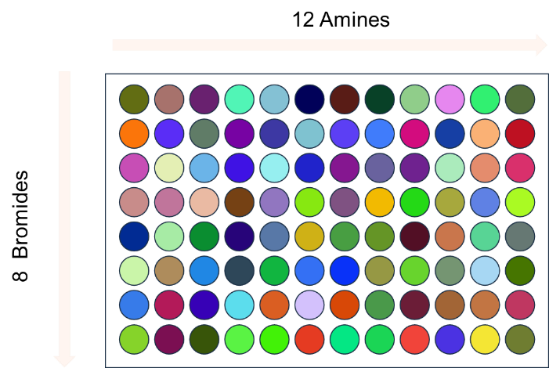


### Figure S1. General reaction setup.

### Table S1. Detailed layout for each plate

| plate | column (amines) | row (bromides) | group |
| --- | --- | --- | --- |
| 1 | a01-a12 | b01-b08 | G1 |
| 2 | a13-a24 | b01-b08 | G1 |
| 3 | a01-a12 | b09-b16 | G1 |
| 4 | a13-a24 | b09-b16 | G1 |
| 5 | a01-a12 | b17-b24 | G1 |
| 6 | a13-a24 | b17-b24 | G1 |
| 7 | a25-a36 | b01-b08 | G2 |
| 8 | a37-a48 | b01-b08 | G2 |
| 9 | a25-a36 | b09-b16 | G2 |
| 10 | a37-a48 | b09-b16 | G2 |
| 11 | a25-a36 | b17-b24 | G2 |
| 12 | a37-a48 | b17-b24 | G2 |
| 13 | a01, a50, a03-a12 | b25-b32 | G3 |
| 14 | a13-a18, a49, a20-a24 | b25-b32 | G3 |
| 15 | a01, a50, a03-a12 | b33-b40 | G3 |
| 16 | a13-a18, a49, a20-a24 | b33-b40 | G3 |
| 17 | a01, a50, a03-a12 | b41-b48 | G3 |
| 18 | a13-a18, a49, a20-a24 | b41-b48 | G3 |
| 19 | a25-a36 | b25-b32 | G4 |
| 20 | a37-a48 | b25-b32 | G4 |
| 21 | a25-a36 | b33-b40 | G4 |
| 22 | a37-a48 | b33-b40 | G4 |
| 23 | a25-a36 | b41-b48 | G4 |
| 24 | a37-a48 | b41-b48 | G4 |

## 1.5 Reaction workup

After reaction, the mixture was cooled to room temperature. The plate was opened and internal standard was added to each well (400 μL of 0.0025 M acetanilide solution in MeCN). The internal standard we used is 4,4'-di-tert-butylbiphenyl (Chart S3). After the solution was fully shaken, the solution was diluted 10 times and sampled into 96-well plates and injected by UPLC.

### Chart S3. Structure of internal standard.

## 1.6 Data analysis

Raw data generated by UPLC-MS were fed to an analytical software developed by Elsci, Peaksel. Official introduction and tutorials for Peaksel are available on <https://www.elsci.io/peaksel/> .

Data was analyzed automatically by Peaksel or manually checked by our chemists. Firstly, the injection name, products’ SMILES and internal standard (IS) SMILES were prepared in *.csv* file. Peaksel would generate substances’ *m/z* accordingly. Then, the corresponding peaks were tracked under mass spectrum and the UV area of substances were integrated under 254±2nm wavelength. Integrated methods were set following the official tutorials in <https://www.elsci.io/peaksel/doc/peaks.html> . We collected data with clearly visible UV peaks (Figure S2) and removed data with overlapping peaks, tailing peaks, splitting peaks and *etc.* (Figure S3). An example for integrated spectra of product and IS was shown in Figure S5. Finally, the ultimate analyzed data with the injection name, products’ area, IS area and ${ratio}_{UV}$ were output in supplementary excel file.


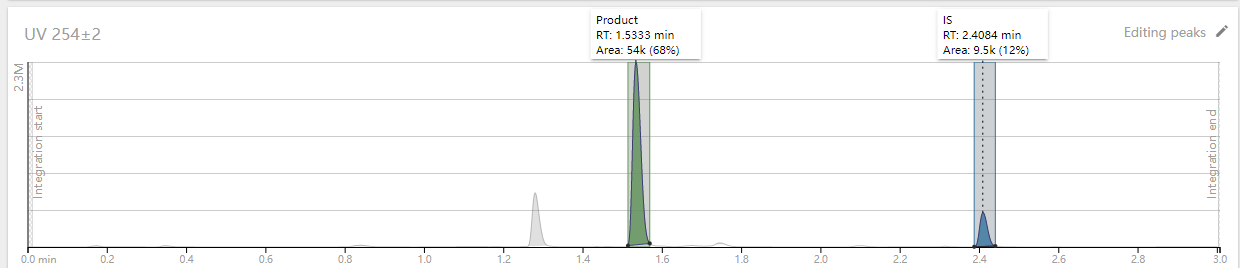


### Figure S2. Example for integrated spectra of product and IS with clearly visible UV peaks under 254±2nm.


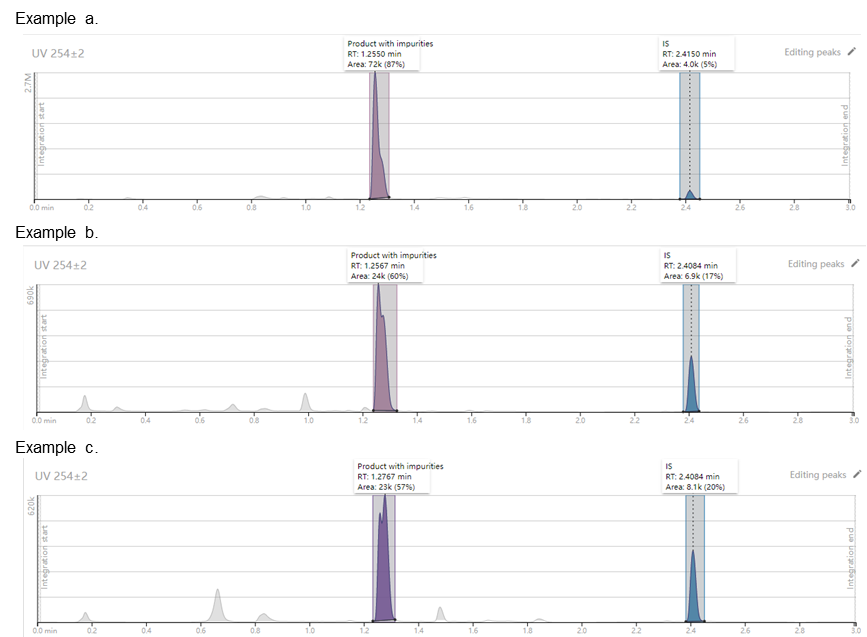


### Figure S3. Examples for integrated spectra with overlapping peaks, tailing peaks, or splitting peaks under 254±2nm. On these occasions, we then removed the data point.

# 2.Details for modelling process

For this part, all scripts and workflows were executed in python scripts. Among them, Yield-BERT and DeepReac+ were used as baseline models.

## 2.1 GraphRXN

The source code of GraphRXN is available at <https://github.com/jidushanbojue/GraphRXN>. The code of GraphRXN was built, relied on *CMPNN’s* molecular representation module. GraphRXN could handle chemical reactions by aggregating molecular graph (summation and concatenation). The ultimate hyper-parameters were listed in Table S2.

### Table S2. The ultimate hyper-parameters of GraphRXN.

| **Hyper-parameters** | **Description** | **Values** |
| --- | --- | --- |
| hidden_size | Dimension of hidden layers in MPN | 300 |
| depth | message passing steps | 3 |
| dropout | Dropout probability | 0 |
| ffn_num_layers | Number of layers in FFN after MPN encoding | 2 |
| add_temp | Use temperature or not | FALSE |
| reaction_agg_method | Specify the aggregate method | sum/concat |
| batch_size | Batch size | 128 |
| init_lr | Initial learning rate | 0.0001 |
| max_lr | Maximum learning rate | 0.001 |
| final_lr | Final learning rate | 0.0001 |

User could easily reproduce our GraphRXN by using ***python reaction_train.py --data_path train_file --separate_test_path test_file --dataset_type regression --num_folds 5 --gpu 0 --epochs 100 --batch_size 128 --save_dir result_path --metric r2 --reaction_agg_method concat***. In addition, temperature in reaction condition would also be taken into account by turning on the switch (--temp). During validation, MSE was taken as loss function, and the performance monitor logs of the model on validation dataset when training was shown in Figure S4~Figure S7.


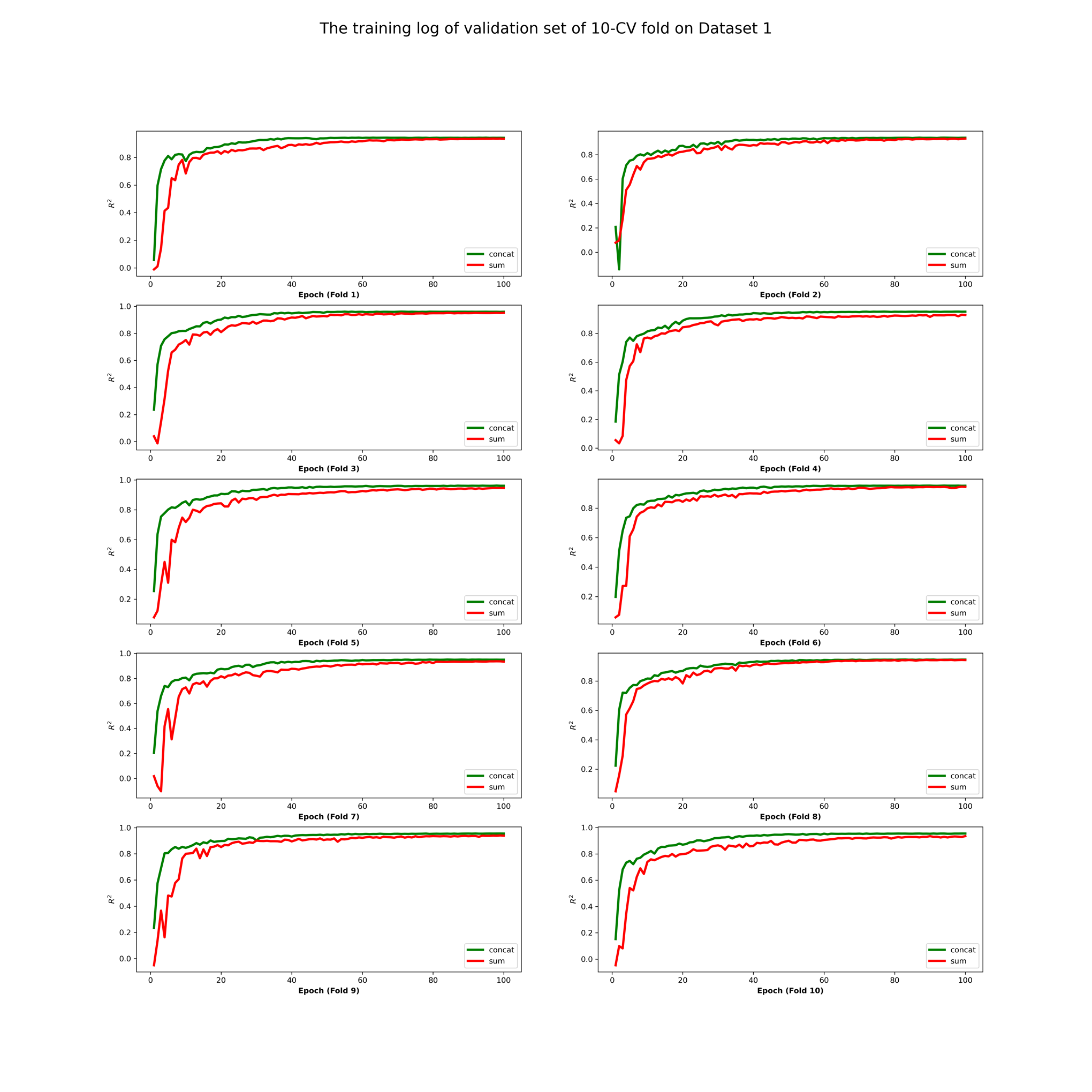


### Figure S4. Training log of validation set of 10-CV on **Dataset 1**.

###
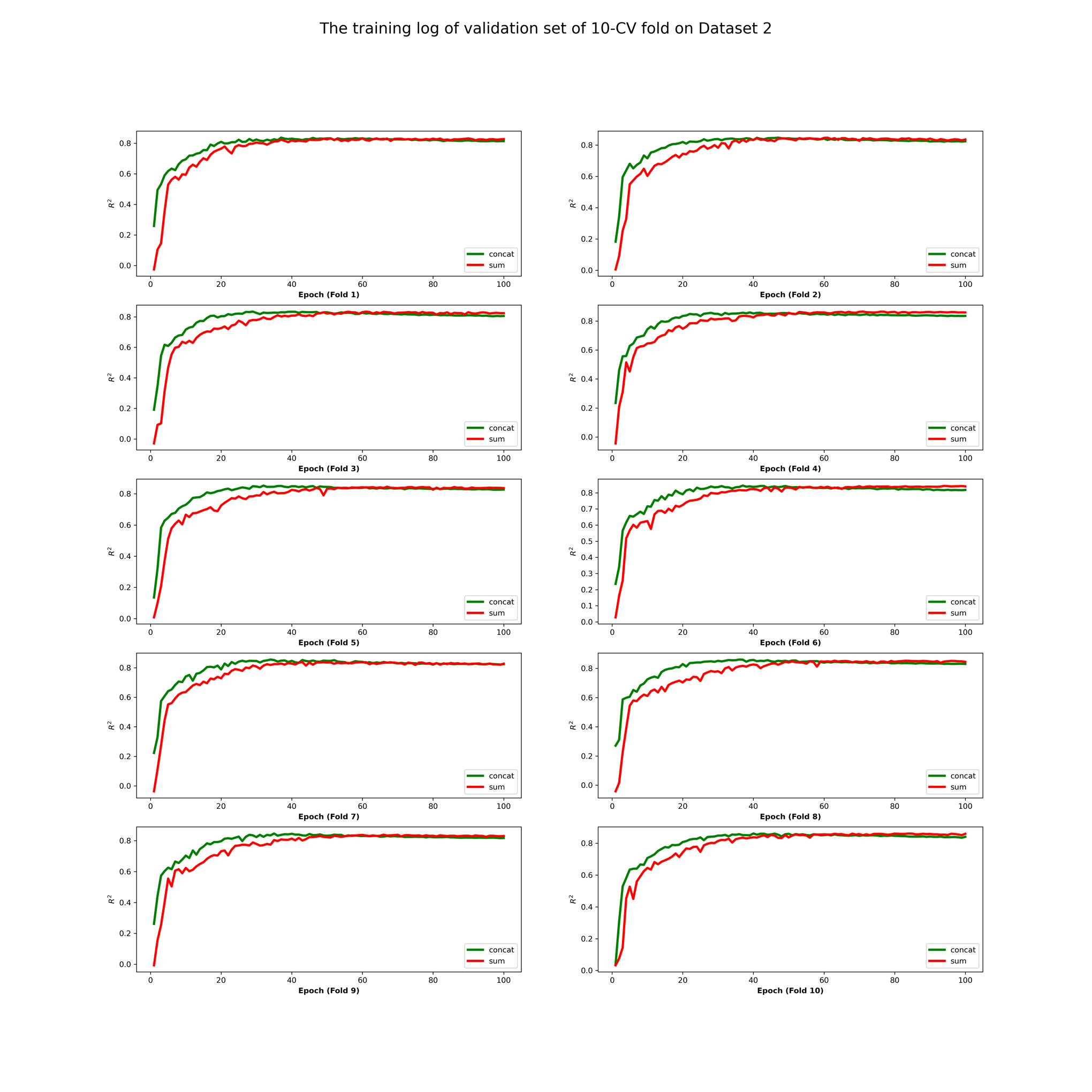
Figure S5. Training log of validation set of 10-CV on **Dataset 2**.


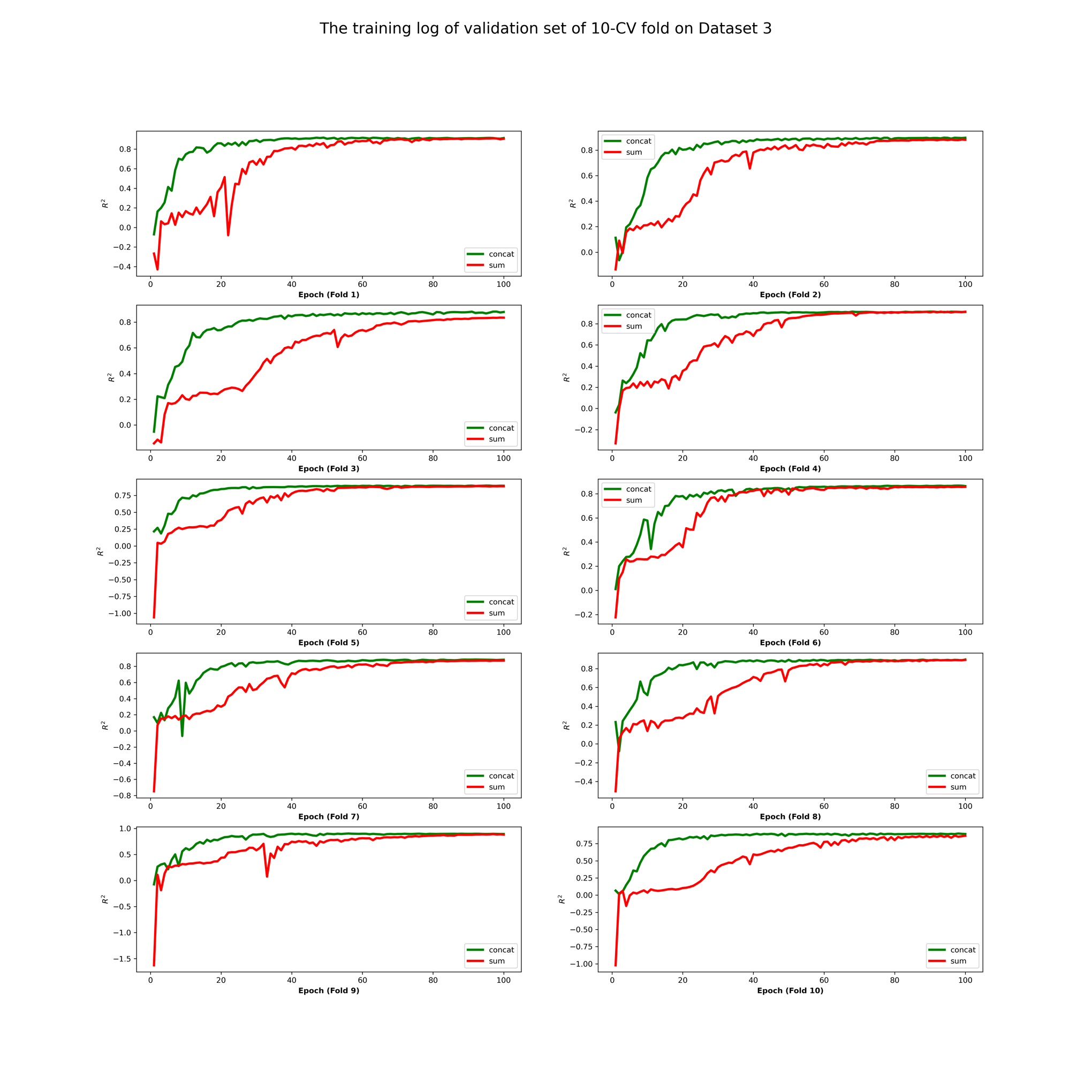


### Figure S6. Training log of validation set of 10-CV on **Dataset 3**.


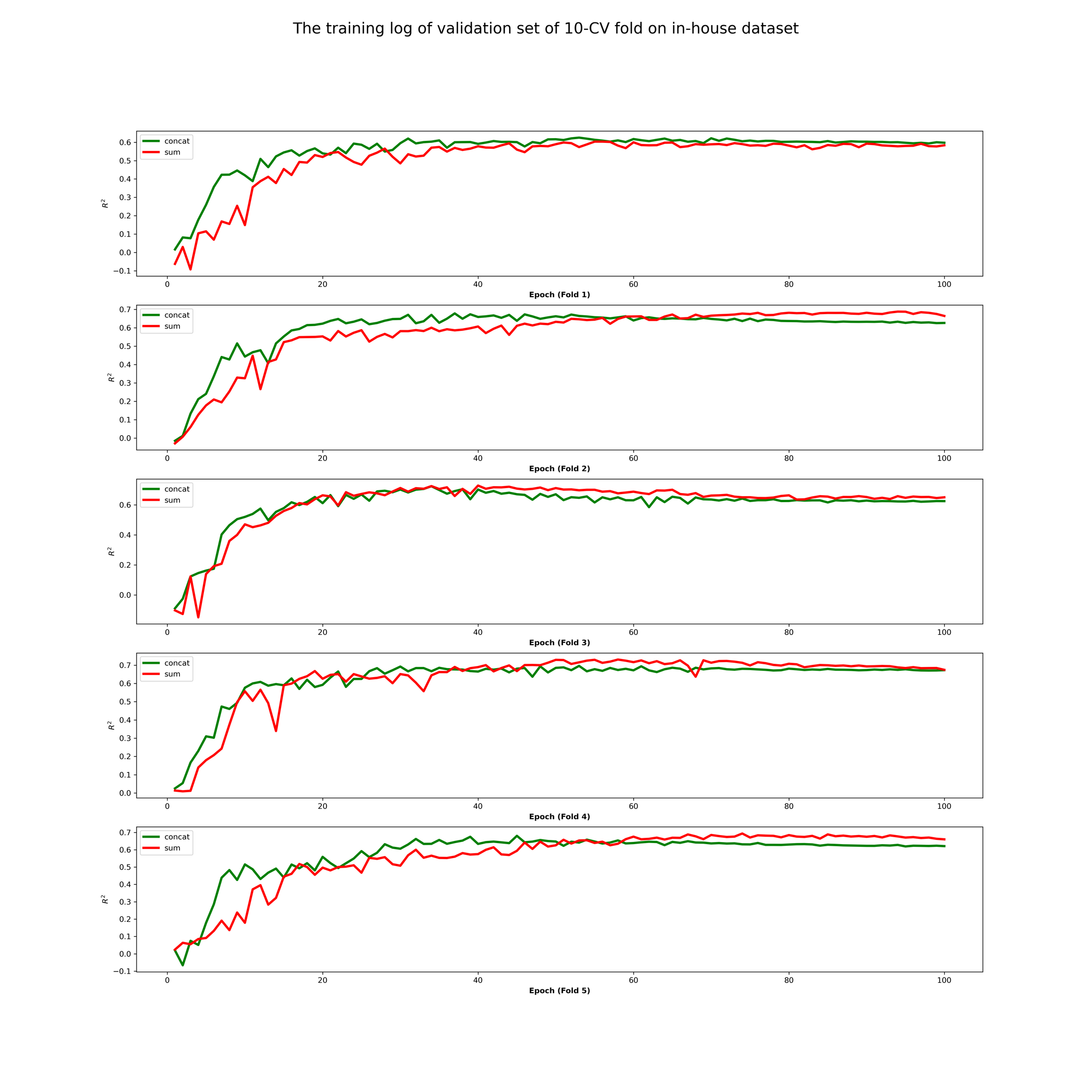


### Figure S7. Training log of validation set of 10-CV on **in-house dataset**.

## 2.2 Yield-BERT

The source code was downloaded from <https://github.com/rxn4chemistry/rxn_yields>. Hyper-parameters searching was done based on dataset 1 using wandb package^1^. The learning rate was sweeping from 1e^-6^ to 1e^-4^, while the dropout rate was sweeping from 0.05 to 0.8, using bayes methods. In this study, the ultimate hyper-parameters used in four datasets were listed in Table S3.

### Table S3. The ultimate hyper-parameters of Yield-BERT.

| **Hyper-parameters** | **Values** |
| --- | --- |
| num_train_epochs | 10 |
| learning rate | 0.00009659 |
| gradient_accumulation_steps | 1 |
| evaluate_during_training | True |
| manual_seed | 42 |
| max_seq_length | 300 |
| train_batch_size | 16 |
| warmup_ratio | 0 |
| hidden_dropout_prob | 0.7987 |

## 2.3 DeepReac+

The source codes for DeepReac+ were downloaded from <https://github.com/bm2-lab/DeepReac>. Some minor modifications were done for resolving some incompatibility issues of python environment, including:

1. Upgrade the DGL version ^2^ from 0.5.2 to 0.8.0.
2. Change the original customized edge-softmax layer to built-in layer.
3. Upgrade routing layer in *Capsule Network* to adapt to the new DGL version instead of the former syntax. This is also the most critical step in a successful operation.

Other important hyper-parameters (such as hidden_feats, num_heads, out_dim, dropout) were come from the DeepReac+ (listed in Table S4). After modification, the DeepReac+ codebase was upload in <https://github.com/jidushanbojue/GraphRXN>.

### Table S4. The ultimate hyper-parameters of DeepReac+

| **Hyper-parameters** | **Description** | **Values** |
| --- | --- | --- |
| in_feats_dim | Input features of each molecule | 74 |
| hidden_feats | Dimension of hidden layers in first GAT layer | [32, 32] |
| num_heads | Number of heads in first GAT layer | [4, 4] |
| hidden_feats | Dimension of hidden layers in second GAT layer | [32, 32] |
| num_heads | Number of heads in second GAT layer | [4, 4] |
| batch_size | Batch size | 128 |

## 2.4 Comparison of training details across four models

Table S5. Comparison of Run Time, Model Size and Epochs

| **Model** | **Run Time (/hour)** | | | **Model Size**  **(Number of Parameters)** | **Epochs** |
| --- | --- | --- | --- | --- | --- |
|  | **Dataset 1** | **Dataset 2** | **Dataset 3** |  |  |
| **GraphRXN-concat** | 8.3 | 10.2 | 0.7 | 2,562,901 | 100 |
| **GraphRXN-sum** | 8.6 | 10.1 | 0.7 | 2,022,901 | 100 |
| **Yield-BERT** | 2.1 | 2.7 | 0.3 | 6,674,689 | 10 |
| **DeepReac+** | 1.6 | 1.6 | 0.2 | 108,130 | 100 |

Note：The models were trained on a single CPU (Inter Xeon 4.00 GHz) with a single GPU (NVIDIA GeForce RTX 2080Ti) on a Linux machine with 64 GB memory

# 3.Details for model performance

## 3.1 Prediction performance on public datasets

Tables S6-S8 show the results on the test set evaluated for each CV fold.

### Table S6. Results of 10-CV folds on test set (Dataset 1)

| Dataset 1 | GraphRXN-conca | | | GraphRXN-sum | | | YieldBERT | | | DeepReac+ | | |
| --- | --- | --- | --- | --- | --- | --- | --- | --- | --- | --- | --- | --- |
| CV | R^2^ | MAE | RMSE | R^2^ | MAE | RMSE | R^2^ | MAE | RMSE | R^2^ | MAE | RMSE |
| 1 | 0.945 | 4.6 | 6.5 | 0.935 | 5.1 | 7.0 | 0.949 | 4.2 | 6.2 | 0.929 | 4.9 | 7.3 |
| 2 | 0.944 | 4.5 | 6.3 | 0.932 | 4.9 | 7.0 | 0.942 | 4.2 | 6.5 | 0.894 | 6.1 | 8.7 |
| 3 | 0.953 | 4.3 | 5.9 | 0.939 | 4.8 | 6.7 | 0.943 | 4.2 | 6.5 | 0.941 | 4.6 | 6.6 |
| 4 | 0.952 | 4.2 | 6.0 | 0.933 | 5.0 | 7.0 | 0.955 | 3.7 | 5.8 | 0.930 | 5.1 | 7.2 |
| 5 | 0.959 | 4.2 | 5.6 | 0.939 | 5.0 | 6.8 | 0.954 | 4.0 | 6.0 | 0.936 | 5.0 | 7.0 |
| 6 | 0.950 | 4.3 | 6.1 | 0.949 | 4.5 | 6.1 | 0.955 | 3.8 | 5.7 | 0.936 | 4.7 | 6.9 |
| 7 | 0.953 | 4.4 | 5.9 | 0.942 | 4.9 | 6.6 | 0.955 | 4.0 | 5.8 | 0.924 | 5.3 | 7.5 |
| 8 | 0.950 | 4.3 | 6.0 | 0.948 | 4.5 | 6.2 | 0.949 | 4.0 | 6.1 | 0.920 | 5.3 | 7.6 |
| 9 | 0.950 | 4.4 | 6.1 | 0.932 | 5.1 | 7.2 | 0.956 | 4.0 | 5.8 | 0.880 | 6.6 | 9.5 |
| 10 | 0.952 | 4.2 | 6.0 | 0.936 | 4.8 | 7.0 | 0.954 | 3.9 | 5.9 | 0.934 | 4.9 | 7.1 |
| average | **0.951** | **4.3** | **6.0** | **0.938** | **4.9** | **6.8** | **0.951** | **4.0** | **6.0** | **0.922** | **5.2** | **7.5** |

### Table S7. Results of 10-CV folds on test set (Dataset 2)

| Dataset 2 | GraphRXN-conca | | | GraphRXN-sum | | | YieldBERT | | | DeepReac+ | | |
| --- | --- | --- | --- | --- | --- | --- | --- | --- | --- | --- | --- | --- |
| CV | R^2^ | MAE | RMSE | R^2^ | MAE | RMSE | R^2^ | MAE | RMSE | R^2^ | MAE | RMSE |
| 1 | 0.846 | 7.9 | 10.9 | 0.837 | 8.0 | 11.2 | 0.808 | 8.3 | 12.2 | 0.830 | 8.0 | 11.5 |
| 2 | 0.843 | 8.1 | 11.1 | 0.840 | 8.1 | 11.3 | 0.831 | 7.8 | 11.6 | 0.839 | 7.8 | 11.3 |
| 3 | 0.846 | 7.9 | 11.0 | 0.855 | 7.8 | 10.7 | 0.839 | 7.4 | 11.3 | 0.848 | 7.6 | 11.0 |
| 4 | 0.852 | 7.7 | 10.8 | 0.836 | 8.1 | 11.3 | 0.821 | 7.9 | 11.9 | 0.828 | 8.3 | 11.6 |
| 5 | 0.858 | 7.8 | 10.5 | 0.849 | 7.8 | 10.8 | 0.817 | 8.1 | 11.9 | 0.852 | 7.5 | 10.8 |
| 6 | 0.845 | 7.8 | 11.0 | 0.832 | 8.2 | 11.4 | 0.805 | 8.6 | 12.3 | 0.834 | 7.7 | 11.4 |
| 7 | 0.839 | 8.0 | 11.5 | 0.824 | 8.6 | 12.0 | 0.807 | 8.5 | 12.6 | 0.827 | 7.9 | 11.9 |
| 8 | 0.848 | 8.0 | 10.8 | 0.843 | 8.1 | 11.0 | 0.822 | 7.9 | 11.7 | 0.799 | 8.8 | 12.5 |
| 9 | 0.834 | 8.2 | 11.6 | 0.836 | 8.1 | 11.5 | 0.796 | 8.5 | 12.8 | 0.816 | 8.3 | 12.2 |
| 10 | 0.833 | 8.1 | 11.5 | 0.831 | 7.9 | 11.5 | 0.802 | 8.3 | 12.5 | 0.801 | 8.7 | 12.5 |
| average | **0.844** | **7.9** | **11.1** | **0.838** | **8.1** | **11.3** | **0.815** | **8.1** | **12.1** | **0.827** | **8.1** | **11.7** |

### Table S8. Results of 10-CV folds on test set (Dataset 3)

| Dataset 1 | GraphRXN-conca | | | GraphRXN-sum | | | YieldBERT | | | DeepReac+ | | |
| --- | --- | --- | --- | --- | --- | --- | --- | --- | --- | --- | --- | --- |
| CV | R^2^ | MAE | RMSE | R^2^ | MAE | RMSE | R^2^ | MAE | RMSE | R^2^ | MAE | RMSE |
| 1 | 0.893 | 0.16 | 0.22 | 0.890 | 0.16 | 0.22 | 0.889 | 0.16 | 0.22 | 0.841 | 0.17 | 0.24 |
| 2 | 0.886 | 0.15 | 0.23 | 0.876 | 0.17 | 0.24 | 0.873 | 0.16 | 0.24 | 0.879 | 0.16 | 0.22 |
| 3 | 0.898 | 0.16 | 0.23 | 0.877 | 0.18 | 0.25 | 0.899 | 0.16 | 0.23 | 0.887 | 0.17 | 0.23 |
| 4 | 0.909 | 0.16 | 0.22 | 0.913 | 0.15 | 0.21 | 0.901 | 0.16 | 0.23 | 0.883 | 0.17 | 0.23 |
| 5 | 0.881 | 0.16 | 0.23 | 0.871 | 0.17 | 0.24 | 0.872 | 0.17 | 0.24 | 0.821 | 0.19 | 0.27 |
| 6 | 0.893 | 0.17 | 0.23 | 0.891 | 0.17 | 0.23 | 0.889 | 0.16 | 0.23 | 0.866 | 0.17 | 0.23 |
| 7 | 0.882 | 0.17 | 0.24 | 0.867 | 0.18 | 0.26 | 0.871 | 0.17 | 0.25 | 0.858 | 0.17 | 0.25 |
| 8 | 0.892 | 0.16 | 0.22 | 0.879 | 0.17 | 0.24 | 0.885 | 0.16 | 0.23 | 0.844 | 0.19 | 0.26 |
| 9 | 0.892 | 0.17 | 0.23 | 0.877 | 0.19 | 0.25 | 0.889 | 0.16 | 0.23 | 0.835 | 0.20 | 0.27 |
| 10 | 0.892 | 0.18 | 0.25 | 0.867 | 0.20 | 0.28 | 0.889 | 0.17 | 0.25 | 0.821 | 0.19 | 0.28 |
| average | **0.892** | **0.16** | **0.23** | **0.881** | **0.18** | **0.24** | **0.886** | **0.16** | **0.24** | **0.853** | **0.18** | **0.25** |

## 3.2 Prediction performance on in-house dataset

A five-fold cross validation without replacement was done for train-test split. Table S9-S13 shows the results evaluated by each CV fold on test set.

### Table S9. Results of 5-CV folds on test set (entire)

| entire | GraphRXN-conca | | | GraphRXN-sum | | | YieldBERT | | | DeepReac+ | | |
| --- | --- | --- | --- | --- | --- | --- | --- | --- | --- | --- | --- | --- |
| CV | R^2^ | MAE | RMSE | R^2^ | MAE | RMSE | R^2^ | MAE | RMSE | R^2^ | MAE | RMSE |
| 1 | 0.708 | 0.07 | 0.09 | 0.683 | 0.07 | 0.09 | 0.644 | 0.07 | 0.10 | 0.644 | 0.07 | 0.10 |
| 2 | 0.741 | 0.07 | 0.09 | 0.735 | 0.07 | 0.09 | 0.674 | 0.07 | 0.10 | 0.649 | 0.07 | 0.10 |
| 3 | 0.731 | 0.06 | 0.08 | 0.730 | 0.06 | 0.08 | 0.667 | 0.06 | 0.09 | 0.550 | 0.07 | 0.10 |
| 4 | 0.706 | 0.06 | 0.08 | 0.686 | 0.06 | 0.08 | 0.651 | 0.06 | 0.09 | 0.595 | 0.07 | 0.09 |
| 5 | 0.674 | 0.07 | 0.10 | 0.675 | 0.07 | 0.10 | 0.582 | 0.07 | 0.11 | 0.580 | 0.08 | 0.11 |

### Table S10. Results of 5-CV folds on test set (G1)

| G1 | GraphRXN-conca | | | GraphRXN-sum | | | YieldBERT | | | DeepReac+ | | |
| --- | --- | --- | --- | --- | --- | --- | --- | --- | --- | --- | --- | --- |
| CV | R^2^ | MAE | RMSE | R^2^ | MAE | RMSE | R^2^ | MAE | RMSE | R^2^ | MAE | RMSE |
| 1 | 0.711 | 0.07 | 0.10 | 0.632 | 0.08 | 0.11 | 0.741 | 0.07 | 0.09 | 0.729 | 0.07 | 0.09 |
| 2 | 0.486 | 0.10 | 0.13 | 0.188 | 0.13 | 0.16 | 0.576 | 0.07 | 0.12 | 0.417 | 0.10 | 0.14 |
| 3 | 0.711 | 0.09 | 0.11 | 0.494 | 0.12 | 0.14 | 0.735 | 0.07 | 0.10 | 0.665 | 0.08 | 0.12 |
| 4 | 0.672 | 0.08 | 0.12 | 0.458 | 0.11 | 0.15 | 0.734 | 0.07 | 0.11 | 0.480 | 0.10 | 0.15 |
| 5 | 0.684 | 0.08 | 0.10 | 0.494 | 0.10 | 0.13 | 0.775 | 0.06 | 0.09 | 0.431 | 0.10 | 0.14 |

### Table S11. Results of 5-CV folds on test set (G2)

| G2 | GraphRXN-conca | | | GraphRXN-sum | | | YieldBERT | | | DeepReac+ | | |
| --- | --- | --- | --- | --- | --- | --- | --- | --- | --- | --- | --- | --- |
| CV | R^2^ | MAE | RMSE | R^2^ | MAE | RMSE | R^2^ | MAE | RMSE | R^2^ | MAE | RMSE |
| 1 | 0.654 | 0.05 | 0.07 | 0.633 | 0.06 | 0.07 | 0.541 | 0.06 | 0.08 | 0.568 | 0.06 | 0.08 |
| 2 | 0.614 | 0.05 | 0.07 | 0.574 | 0.06 | 0.08 | 0.488 | 0.06 | 0.08 | 0.547 | 0.06 | 0.08 |
| 3 | 0.577 | 0.05 | 0.07 | 0.565 | 0.06 | 0.08 | 0.469 | 0.06 | 0.08 | 0.548 | 0.06 | 0.08 |
| 4 | 0.708 | 0.04 | 0.06 | 0.628 | 0.05 | 0.07 | 0.587 | 0.05 | 0.07 | 0.546 | 0.06 | 0.07 |
| 5 | 0.589 | 0.05 | 0.07 | 0.549 | 0.05 | 0.07 | 0.471 | 0.05 | 0.08 | 0.406 | 0.06 | 0.08 |

### Table S12. Results of 5-CV folds on test set (G3)

| G3 | GraphRXN-conca | | | GraphRXN-sum | | | YieldBERT | | | DeepReac+ | | |
| --- | --- | --- | --- | --- | --- | --- | --- | --- | --- | --- | --- | --- |
| CV | R^2^ | MAE | RMSE | R^2^ | MAE | RMSE | R^2^ | MAE | RMSE | R^2^ | MAE | RMSE |
| 1 | 0.832 | 0.05 | 0.07 | 0.780 | 0.06 | 0.08 | 0.780 | 0.06 | 0.08 | 0.732 | 0.07 | 0.09 |
| 2 | 0.747 | 0.06 | 0.08 | 0.735 | 0.06 | 0.08 | 0.771 | 0.05 | 0.08 | 0.769 | 0.06 | 0.08 |
| 3 | 0.820 | 0.05 | 0.07 | 0.791 | 0.05 | 0.07 | 0.770 | 0.05 | 0.08 | 0.746 | 0.06 | 0.08 |
| 4 | 0.813 | 0.06 | 0.07 | 0.780 | 0.06 | 0.08 | 0.802 | 0.06 | 0.08 | 0.690 | 0.07 | 0.10 |
| 5 | 0.788 | 0.06 | 0.08 | 0.779 | 0.06 | 0.08 | 0.791 | 0.06 | 0.08 | 0.782 | 0.07 | 0.08 |

### Table S13. Results of 5-CV folds on test set (G4)

| G4 | GraphRXN-conca | | | GraphRXN-sum | | | YieldBERT | | | DeepReac+ | | |
| --- | --- | --- | --- | --- | --- | --- | --- | --- | --- | --- | --- | --- |
| CV | R^2^ | MAE | RMSE | R^2^ | MAE | RMSE | R^2^ | MAE | RMSE | R^2^ | MAE | RMSE |
| 1 | 0.450 | 0.08 | 0.11 | 0.314 | 0.09 | 0.12 | 0.515 | 0.08 | 0.10 | 0.057 | 0.11 | 0.15 |
| 2 | 0.604 | 0.07 | 0.10 | 0.514 | 0.08 | 0.11 | 0.566 | 0.08 | 0.11 | 0.513 | 0.08 | 0.11 |
| 3 | 0.410 | 0.10 | 0.14 | 0.512 | 0.09 | 0.12 | 0.491 | 0.09 | 0.13 | 0.104 | 0.11 | 0.17 |
| 4 | 0.335 | 0.08 | 0.10 | 0.374 | 0.08 | 0.10 | 0.398 | 0.07 | 0.10 | 0.092 | 0.10 | 0.12 |
| 5 | 0.426 | 0.08 | 0.13 | 0.311 | 0.10 | 0.14 | 0.480 | 0.08 | 0.12 | 0.273 | 0.10 | 0.15 |

## 3.3 Model performance under different vector sizes

Several exploratory experiments were conducted to test whether the vector size affect our model performance. The detailed metrics were listed in Table S14-S15.

### Table S14. Model performance of GraphRXN-concat under the vector sizes from 100 to 900 bits.

| Vector size | R^2^ | MAE | RMSE |
| --- | --- | --- | --- |
| 100 | 0.701 ± 0.022 | 0.06 ± 0 | 0.09 ± 0 |
| 200 | 0.710 ± 0.0240 | 0.06 ± 0 | 0.09 ± 0 |
| 300 | 0.712 ± 0.023 | 0.06 ± 0 | 0.09 ± 0 |
| 400 | 0.703 ± 0.029 | 0.06 ± 0 | 0.09 ± 0 |
| 500 | 0.706 ± 0.031 | 0.06 ± 0 | 0.09 ± 0 |
| 600 | 0.703 ± 0.035 | 0.06 ± 0 | 0.09 ± 0 |
| 700 | 0.697 ± 0.044 | 0.06 ± 0 | 0.09 ± 0 |
| 800 | 0.699 ± 0.034 | 0.06 ± 0 | 0.09 ± 0 |
| 900 | 0.697 ± 0.031 | 0.06 ± 0 | 0.09 ± 0 |

### Table S15. Model performance of GraphRXN-sum under the vector sizes from 100 to 900 bits.

| Vector size | R^2^ | MAE | RMSE |
| --- | --- | --- | --- |
| 100 | 0.685 ± 0.014 | 0.07 ± 0 | 0.09 ± 0 |
| 200 | 0.693 ± 0.021 | 0.06 ± 0 | 0.09 ± 0 |
| 300 | 0.702 ± 0.025 | 0.06 ± 0 | 0.09 ± 0 |
| 400 | 0.697 ± 0.020 | 0.06 ± 0 | 0.09 ± 0 |
| 500 | 0.697 ± 0.020 | 0.06 ± 0 | 0.09 ± 0 |
| 600 | 0.693 ± 0.023 | 0.06 ± 0 | 0.09 ± 0 |
| 700 | 0.697 ± 0.025 | 0.06 ± 0 | 0.09 ± 0 |
| 800 | 0.691 ± 0.032 | 0.06 ± 0 | 0.09 ± 0 |
| 900 | 0.695 ± 0.026 | 0.06 ± 0 | 0.09 ± 0 |

# 4. Reference

1 Biewald, L. Experiment tracking with weights and biases. *Software available from wandb. com* **2** (2020).

2 Wang, M. *et al.* Deep graph library: A graph-centric, highly-performant package for graph neural networks. *arXiv preprint arXiv:1909.01315* (2019).
